# Engineering *Synechocystis* PCC6803 for Hydrogen Production: Influence on the Tolerance to Oxidative and Sugar Stresses

**DOI:** 10.1371/journal.pone.0089372

**Published:** 2014-02-24

**Authors:** Marcia Ortega-Ramos, Thichakorn Jittawuttipoka, Panatda Saenkham, Aurelia Czarnecka-Kwasiborski, Hervé Bottin, Corinne Cassier-Chauvat, Franck Chauvat

**Affiliations:** 1 UMR8221, CEA, CNRS, Université Paris Sud, Institut de Biologie et Technologie Saclay, Laboratoire de Biologie et Biotechnologie des Cyanobactéries, CEA-Saclay, Gif sur Yvette, France; 2 UMR8221, CEA, CNRS, Université Paris Sud, Institut de Biologie et Technologie Saclay, Laboratoire des Mécanismes fondamentaux de la Bioénergétique, CEA-Saclay, Gif sur Yvette, France; University of Florida, United States of America

## Abstract

In the prospect of engineering cyanobacteria for the biological photoproduction of hydrogen, we have studied the hydrogen production machine in the model unicellular strain *Synechocystis* PCC6803 through gene deletion, and overexpression (constitutive or controlled by the growth temperature). We demonstrate that the hydrogenase-encoding *hoxEFUYH* operon is dispensable to standard photoautotrophic growth in absence of stress, and it operates in cell defense against oxidative (H_2_O_2_) and sugar (glucose and glycerol) stresses. Furthermore, we showed that the simultaneous over-production of the proteins HoxEFUYH and HypABCDE (assembly of hydrogenase), combined to an increase in nickel availability, led to an approximately 20-fold increase in the level of active hydrogenase. These novel results and mutants have major implications for those interested in hydrogenase, hydrogen production and redox metabolism, and their connections with environmental conditions.

## Introduction

Cyanobacteria are the only known prokaryotes capable of oxygenic photosynthesis, which uses nature's most abundant resources, solar energy, water, CO_2_ and mineral nutrients, to produce a large part of the oxygen and organic assimilates for the aerobic food chain. Furthermore, cyanobacteria are regarded as promising “low-cost” microbial cell factories for carbon capture and storage, and the sustainable production of biofuels, thanks to their simple nutritional requirements; their physiological robustness (in colonizing a wealth of biotopes they will enable future industrial productions to be performed near the sites of use, to reduce transportation costs); and the powerful genetics of some model strains. One of the fuels of special interest is hydrogen (H_2_) because it is a high-energy fuel [1] that burns cleanly in producing only water as its by-product. Thus, it is important to investigate the cyanobacterial machine for the production of hydrogen. The complex cyanobacterial machine for hydrogen production (Figure S1) is best studied in the widely used unicellular strain *Synechocystis* PCC6803 [2] (hereafter designated as *Synechocystis*), which harbours a small genome (less than 4 Mb; See CyanoBase: http://genome.kazusa.or.jp/cyanobase/) easily manipulable [3]-[6]. The pentameric hydrogenase enzyme (HoxEFUYH; Hox for hydrogen oxidation), which is reversibly inactivated by oxygen [7], is a bidirectional enzyme with a bias to H_2_ production [8]. This reaction, 2H^+^ + 2e^−^ ↔ H_2_, uses NAD(P)H as the source of electrons originating from photosynthesis and/or sugar catabolism, and a nickel-iron center and several iron-sulfur clusters as redox cofactors [9]. The five *Synechocystis* genes *hoxEFUYH* are clustered in an octacistronic operon that comprises the following genes *hoxE*, *hoxF*, *sll1222, hoxU*, *hoxY*, *ssl2420*, *sll1225* and *hoxH* in that order, which also encode the three proteins of unknown function Sll1222, Ssl2420 and Sll1225 [2], [9]. The *hoxEFUYH* operon is expressed by a weak promoter [10], which generates a polycistronic transcript that initiates 168 bp upstream of the start codon of the proximal *hoxE* gene [11], [12]. The HoxEFU subunits make up the diaphorase sub-complex that transfers the electrons provided by NAD(P)H [7] to the hydrogenase unit HoxHY that reduces the protons to generate H_2_ (Figure S1). After HoxYH assembly, the HoxH subunit is processed by the HoxW protease [2]. The Hox complex is assembled by the six-subunits HypABCDEF complex enzyme [2], [9] encoded by the *hypABCDEF* genes, which are scattered onto the chromosome (Figure S1). Physiological studies indicated that the hydrogenase enzyme acts as an emergency electron valve to release excess of photosynthetic electrons, for instance during the transition from (anaerobic) dark to light conditions, leading to weak and transient H_2_ production [7], [9].

To attempt increasing hydrogenase activity in *Synechocystis* Germer and co-workers have used the light-inducible promoter of the photosynthetic gene *psbAII* to increase the expression of the endogenous *hoxEFUYH* operon and the heterologous _Nos_ *hypABCDEF* operon from *Nostoc* PCC7120 [13]. The gain in activity was modest (3.2 fold; *i.e.* from 2.9 nmol H_2_.min^-1^.mg chlorophyll^−1^ in wild-type cells up to 9.4 nmol H_2_.min^−1^.mg chlorophyll^−1^ in mutant cells) for the following possible reasons. First, the light-inducible *psbAII* promoter used to increase the expression of the *hoxEFUYH* and the _Nos_ *hypABCDEF* genes is more active under high light, which increases the photosynthetic production of O_2_, which inhibits hydrogenase activity. Second, the C-terminus of the HoxF subunit was fused to the strep tag, which might have interfererred with HoxF activity. Third, the Km^r^ marker gene that was introduced downstream of the strep-tagged *hoxF* gene might have decreased the expression of the downstream genes *hoxUYH*, at least in increasing the spacing distance between them and the *psbAII* promoter. Fourth, it is also possible that the *Nostoc* HypABCDEF proteins are not fully active on the *Synechocystis* PCC6803 HoxEFUYH proteins, or could somehow interfere with hydrogen production (for instance interfere with the function of the endogenous *Synechocystis* HypABCDEF proteins). Fifth, the *_nos_hypABCDEF* operon contains a gene, *asr0697* (located between *hypD* and *hypE* [14]) encoding a protein (a putative 4-oxalocrotonate tautomerase), which is not normally present in *Synechocystis* and could interfere with hydrogen metabolism. For the same objective of increasing the abundance of active hydrogenase we have characterized and deleted the *Synechocystis* AbrB2 repressor of the *hoxEFUYH* operon [10], [15] with a moderate success (two fold increase in the level of active hydrogenase as compared to the wild-type strain).

In spite of these studies, the role of the hydrogen metabolism remains puzzling. A better understanding of this role is required to identify suitable environmental conditions and powerful genetic manipulations to improve the level of hydrogen production. In this prospect, we have studied the hydrogen production machine in the model unicellular strain *Synechocystis* PCC6803 through gene deletion, and overexpression (constitutive or controlled by the growth temperature). We report that the hydrogenase-encoding *hoxEFUYH* operon is dispensable to standard photoautotrophic growth in absence of stress, and it operates in cell defense against oxidative (H_2_O_2_) and sugar (glucose and glycerol) stresses. Furthermore, we show that the simultaneous over-production of the endogenous proteins HoxEFUYH and HypABCDEF, combined to an increase in nickel availability led to an approximately 20-fold increase in active hydrogenase level. These novel results and mutants have major implications for the engineering of effective cyanobacterial factories for the biological production of hydrogen from solar energy.

## Materials and Methods

### Bacterial strains and culture conditions

*Synechocystis* PCC6803 was grown at 30°C or 39°C (depending on the experiments) under continuous white light (standard intensity is 2,500 lux; 31.25 µE m^−2^ s^−1^) on mineral medium (MM), *i.e.* BG11 enriched with 3.78 mM Na_2_CO_3_ [16]. Some cultures were grown in MM supplemented with 17 µM Fe (provided as green ferric ammonium citrate) and 2.5 µM NiSO_4_, for hydrogenase assay, or with 10 mM glucose or 300 µM glycerol, as indicated. For survival analyses, exponentially growing cells were harvested at the density of 2.5×10^7^ cells.ml^−1^, washed and resuspended in MM at the same concentration. Then, 1 ml aliquotes were incubated under anaerobic conditions for 30 min under dim light and for 1 h in darkness with H_2_O_2_ at the indicated concentrations. Cells were washed and resuspended in an equal volume of MM, diluted, spread on MM solidified with 1% agar (difco) and subsequently incubated at 30°C under standard light. Surviving colonies were counted after 5-7 days of growth.

*E. coli* strains used for gene manipulations (TOP10; Invitrogen® or DH5α for conjugative transfer to *Synechocystis* (CM404) of replicative plasmids (Table S1) derived from pFC1 [5] were grown on LB medium at 30°C (CM404 and TOP10 harbouring pFC1 derivatives) or 37°C (TOP10, DH5α). Antibiotic selection was as follows: ampicillin (Ap) 100 µg.ml^−1^, 50 µg.ml^−1^, kanamycin (Km) 50 µg.ml^−1^, and spectinomycin (Sp) 100 µg.ml^−1^ for *E. coli*; Km 50–300 µg.ml^−1^, Sp 2.5–5 µg.ml^−1^ and streptomycin (Sm) 2.5–5 µg.ml^−1^ for *Synechocystis*.

### Construction of pFC1K, the Kmr replicative plasmid vector for temperature-controlled gene expression in *Synechocystis*

pFC1K (Table S1) was constructed (Figure S2) using the vector pFC1, which possesses the λ*c*I_857_-λ*p*_R_ system for temperature-regulated gene expression and replicates autonomously in *E. coli* and cyanobacteria [5]. In *Synechocystis* pFC1 replicates at the same 10–20 copies number per cell as the chromosome [5]. The λ*c*I_857_ temperature sensitive repressor-encoding gene tightly controls the activity of the otherwise strong λ*p*_R_ promoter that is followed by the λ*cro* ribosome-binding site (5′-AGGA-3′) and ATG start codon embedded within the unique *Nde* I restriction site (5′-CAT**ATG**-3′) for in frame-fusion of the protein coding region of the studied genes [5], [10]. The Km^r^ gene was PCR amplified from the commercial pUC4K plasmid (Table S1) with the oligonucleotide primers KmHinCFW and KmHinCRV (Table S2), which introduced a flanking *Hinc*II restriction site. After *Hinc*II cleavage, the Km^r^ gene was cloned in place of the Sm^r^/Sp^r^ marker of pFC1 opened with *Nae*I and *Xmn*I, yielding pFC1K which was verified by PCR and nucleotide sequencing (Big Dye kit, ABI Perking Elmer).

### RNA isolation and analysis

200 ml of mid-log phase cultures (2.5×10^7^cells ml^−1^) were rapidly harvested by vacuum filtration (less than 1 min); resuspended in 4 ml Tris 50 mM pH 8, EDTA 5 mM; immediately frozen in an Eaton press chamber cooled in a dry ice and ethanol bath and disrupted (250 MPa). RNA were extracted and purified with the Qiagen kit RNAeasy as we described [10], [17]. RNA concentration and purity (A_260_/A_280_ >1.9) were determined with a Nanodrop (Thermo scientific) and migration on agarose gel to verify the absence of RNA degradation. The absence of contaminant DNA was verified with the *Taq* DNA-dependent DNA-Polymerase (Invitrogen) using primers specific to the control gene *rnpB* (Table S2). For quantitative RT-PCR the gene specific primers were chosen so as to generate DNA fragment of similar length comprised between 163 bp and 234 bp. Each assay was triplicated, allowing the mean threshold cycle value (*C_T_*) to be calculated from standard curve by the iQ5 optical system software (BioRad). Each gene-specific standard curve was made by 4 fold serial dilution of wild-type strain cDNA (ranging from 9375 to 9.16 ng) against log input cDNA concentration for each primer (data not shown). For each primer tested, the regression value (*DC_T_* versus cDNA concentration) was less than 0.1, indicating approximately equal amplification efficiencies. Then, for each studied gene the *C_T_* value was converted to gene copy number per ng of template cDNA.

### Western blot analysis of the HoxF and HoxH proteins

40 µg of *Synechocystis* proteins separated on 12% SDS PAGE (Thermo scientific) were transferred (iBlot system; Invitrogen) to nitrocellulose membrane (Invitrogen), which were blocked for 1 h at room temperature or overnight at 4°C with 5% non-fat milk in phosphate buffered saline (PBS). Immunodetection was performed using the following rabbit antibodies [18] anti-HoxF (dilution 1∶2000) and anti-HoxH (dilution of 1∶500). R800 goat anti-rabbit IgG (Invitrogen) were used as secondary antibodies (dilution of 1∶1000), and immune complexes were visualized by chemiluminescence (ECL from GE Healthcare Amersham).

### Proteomics experiments

LC-MS/MS analysis and identification of the HoxEFUYH and HypABCDEF proteins were performed on the PAPPSPO platform (http://pappso.inra.fr/index.php?lang=en), using QExactive mass spectrometer (ThermoFinnigan) and/or a LTQ-Orbitrap Discovery (ORBITRAP Discovery; ThermoFinnigan).

### Hydrogenase activity measurements

Hydrogenase activities were measured on 1 ml aliquots of mid-log phase culture concentrated 5.7-fold by centrifugation, using a modified Clark-type electrode (Hansatech, UK), and saturating amounts of Na-dithionite (20 mM) and methylviologen (5 mM) as the electron donor, as we previously described [10].

## Results

### The hoxEFUYH operon is dispensable to the photoautotrophic growth of *Synechocystis*

**Figure 1:**
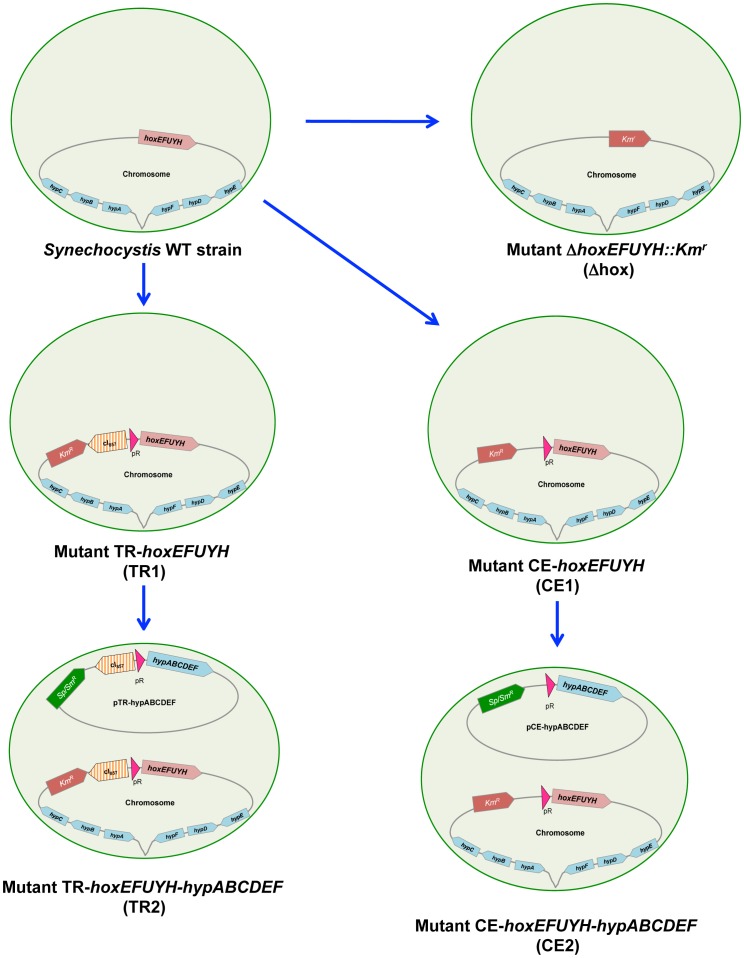
Schematic representation of the *Synechocystis* wild-type and the mutant strains constructed in this study. The *Synechocystis* spherical cells are represented by the green circles. The chromosome is shown as the black line form attached to the cell membrane, while the pTR-*hypABCDEF* and pCE-*hypABCDEF* replicating plasmids are represented by circles. The *hoxEFUYH* operon, the *hypABCDEF* genes and the antibiotic resistance markers are shown by large colored arrows, which indicate the direction of their transcription. The hatched (orange) arrow shows the λ*c*I_857_ gene encoding the temperature-sensitive repressor, which tightly controls the activity of the otherwise strong λ*p*_R_ promoter (red triangle), depending on the growth temperature. The symbols are namely: Δ for deletion; CE for constitutive expression; TR for temperature-regulated expression; and WT for wild type.

To investigate the role of the octacistronic *hoxEFUYH* operon on the physiology of *Synechocystis*, we constructed a Δ*hoxEFUYH*::Km^r^ deletion mutant (Figure 1) by replacing the whole *hoxEFUYH* operon (from 58 bp upstream of the ATG start codon of *hoxE* to 8 bp downstream of the stop codon TAA of *hoxH*) by a Km^r^ marker gene (Figure S3). Therefore, the Km^r^ gene was amplified from the pFC1K plasmid constructed in this study (Figure S2 and Table S1), using specific oligonucleotide PCR primers (Table S2) that generated *Sph*I and *Afl*II restriction sites for cloning into pGEM-T (Figure S3). Meanwhile, the two regions of *Synechocystis* DNA (about 300 bp each) flanking the *hoxEFUYH* operon were independently amplified with PCR primers (Table S2) that generated *Sph*I or *Afl*II restriction sites (Figure S3). After cleavage, these *Synechocystis* DNA segments were cloned on each side of the Km^r^ marker of the pGEM-T derivative to serve as platforms for homologous recombinations promoting targeted gene replacement [19] (*i.e.* replacement of the *hoxEFUYH* operon by the Km^r^ gene) upon transformation. The resulting Δ*hoxEFUYH*::Km^r^ DNA cassette (Figure S3) was verified by PCR and nucleotide sequencing, prior to transformation to *Synechocystis* [19]. Km^r^ mutants were analyzed by PCR (Figure S4) to verify that the Km^r^ marker had properly replaced the whole *hoxEFUYH* operon (from 71 bp upstream the *hoxE* ATG start codon to 19 bp after the *hoxH* TAA stop codon) in all copies of the chromosome, which is polyploïd [19]. The absence of the *hoxEFUYH* operon in the Δ*hoxEFUYH*::Km^r^ mutant was confirmed upon the analysis of culture grown for a few generations in absence of Km to stop counter-selecting the propagation of possibly remaining wild-type (WT) chromosome copies, prior to the PCR assays (Figure S4). We also confirmed through quantitative RT-PCR that the Δ*hoxEFUYH*::Km^r^ mutant completely lacks *hoxEFUYH* transcripts (data not shown), as well as the HoxF and HoxH proteins and hydrogenase activity (Figure S5). These data, together with the fact that the Δ*hoxEFUYH*::Km^r^ mutant grows as healthy as the WT strain under standard photoautotrophic conditions (Figure S5), showed that the *hoxEFUYH* operon is dispensable in *Synechocystis*. This finding is consistent with the previously observed dispensability of the *Synechocystis hoxHY* genes in the otherwise WT strain [20], and the *hoxEFUYH* operon in the glucose tolerant mutant [2].

### Construction and analysis of a mutant for temperature-controlled high-level expression of the *hoxEFUYH* operon: replacement of its weak promoter by the strong promoter λp_R_ controlled by the temperature-sensitive repressor λcI_857_

**Figure 2:**
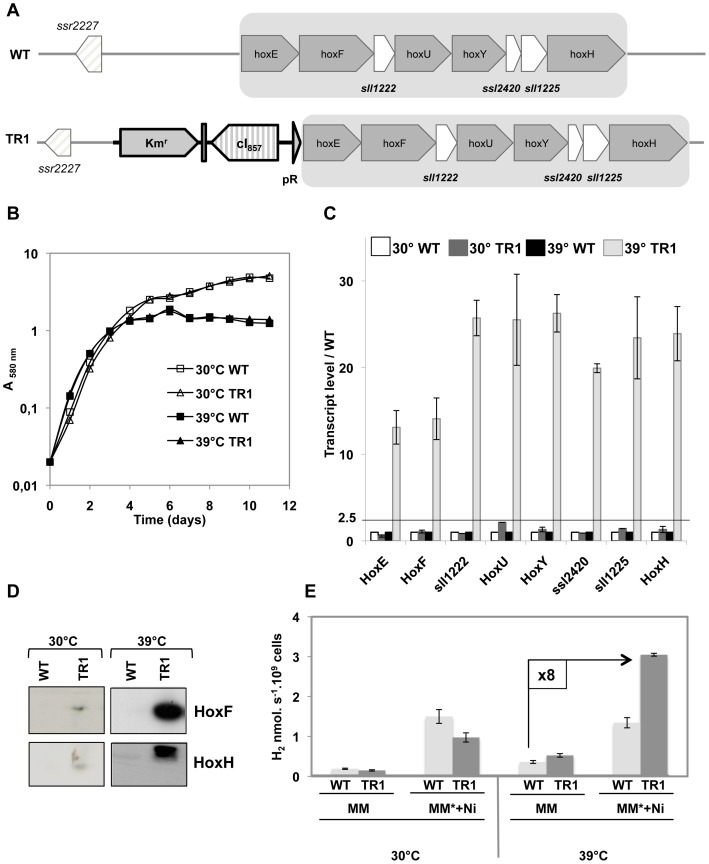
Analysis of the *Synechocystis* TR-*hoxEFUYH* mutant for temperature-regulated high-level expression of the *hoxEFUYH* operon. (**A**) Schematic representation of the *hoxEFUYH* operon locus in the wild-type strain (WT) and the TR-*hoxEFUYH* mutant (TR1). (**B**) Typical growth of the WT (squares) and TR1 cells (triangles) under standard light at 30°C (open symbols) or 39°C (black symbols). (**C**) Histogram representation of the ratio of the transcript abundance (measured by Real-time quantitative PCR) of each eight genes of the *hoxEFUYH* operon in the WT and TR1 cells grown at 30°C or 39°C. (**D**) Western blot analysis of the abundance of the HoxF and HoxH proteins in WT and TR1 cells grown at 30°C or 39°C. (**E**) Histograms representation of the hydrogenase activities of WT and TR1 cells grown at 30°C or 39°C in standard medium (MM) or MM* (MM +17 µM Fe) supplemented with 2.5 µM NiSO_4_. All experiments were performed at least three times.

To increase the expression of the *hoxEFUYH* operon, while caring with the possibility that above a certain level it might become toxic, we decided to replace the weak promoter of the *hoxEFUYH* operon [10] by a strong and controllable promoter. Therefore, we used the λ*c*I_857_-λ*p_R_* system that expresses genes proportionally to the temperature of growth [4], [5], [21]: *i.e.* absence of expression at 30°C (the standard growth temperature) and strong expression at 39°C (good growth of wild-type cells). Practically, the 2.8 kb Km^r^-λ*c*I_857_-λ*p_R_* DNA cassette of the pFC1K plasmid presently constructed (Figure S2) was amplified by PCR amplified, and introduced in place of the *hoxEFUYH* operon promoter (the 691 bp region upstream of the ATG start codon of *hoxE*), as follows (Figure S6). The 252 bp region of *Synechocystis* DNA upstream of the *hoxEFUYH* operon promoter was amplified by PCR with specific oligonucleotide primers (Table S2), digested with the *BamH*I and *Sph*I and cloned upstream of the Km^r^ marker of pFC1K opened with the same enzymes. Meanwhile, the 527 bp region of *Synechocystis* DNA encompassing the *hoxE* coding sequence was cloned as a *Nde*I-*Eco*RI DNA segment downstream of the λ*p*_R_-promoter of pFC1K opened with the same enzymes. The resulting pTR-*hoxEFUYH* plasmid was linearized with *Eco*RV and transformed to *Synechocystis*, where homologous recombinations introduced the Km^r^-λ*c*I_857_-λ*p_R_* DNA cassette in place of the natural promoter of the *hoxEFUYH* operon. Km^r^ transformant clones growing in standard conditions were analyzed by PCR (Figure S7) to verify that the Km^r^-λ*c*I_857_-λ*p_R_* DNA cassette had properly replaced the natural *hoxEFUYH* operon promoter, in all copies of the polyploïd chromosome. We verified that this mutant, hereafter designated as TR-*hoxEFUYH* (Figure 1), possessed no wild-type (WT) chromosome copies, even when cells were grown in absence of Km that otherwise counter-select the propagation of WT chromosome copies (Figure S7). The TR-*hoxEFUYH* mutant strain grew as fit as the WT strain at both 30°C and 39°C (Figure 2). Then, we verified through quantitative RT-PCR analysis that the λ*c*I_857_-λ*p_R_* promoter system expressed the TR-*hoxEFUYH* operon in a true temperature-controlled way. Therefore, total RNAs isolated from the TR-*hoxEFUYH* mutant and WT strains grown at either 30°C or 39°C were hybridized with specific RT-PCR primers (Table S2) designed to amplify an internal segment of each eight genes of the *hoxEFUYH* operon (*hoxE*, *hoxF*, *sll1222*, *hoxU*, *hoxY*, *ssl12420*, *sll1225* and *hoxH*; Figure 1). The abundance of all eight transcripts were similar in WT cells grown at 30°C (the standard temperature) or 39°C (Figure 2), showing that the expression of the WT-*hoxEFUYH* operon from its natural promoter is not affected by these temperatures. By contrast, all eight *hoxEFUYH* mRNAs were much more abundant in TR-*hoxEFUYH* cells grown at 39°C as compared to 30°C, in agreement with our previous reports that at 30°C the λcI_857_ repressor strongly inhibits the activity of the λ*_PR_* promoter [4], [5]. Furthermore, at 39°C the *hoxEFUYH* transcript levels were higher in the TR-*hoxEFUYH* mutant than in the WT strain (Figure 2). Consistently, the abundance of the HoxF and HoxH proteins were higher in TR-*hoxEFUYH* cells grown at 39°C as compared to 30°C, or to WT cells grown either at 30°C or 39°C (Figure 2).

Collectively, these data demonstrated that the *hoxEFUYH* operon is expressed in a temperature-controlled way in the TR-*hoxEFUYH* mutant, and that the overexpression of the *hoxEFUYH* operon at 39°C is not detrimental to cell life. However, the 39°C-induction of the level of active hydrogenase in the TR-*hoxEFUYH* mutant was modest in comparison to the strong induction of the *hoxEFUYH* transcripts or HoxF and HoxH proteins (Figure 2). As the activity of the Ni-Fe hydrogenase can be limited by the availability of Fe and/or Ni atoms [22], [23], we have also measured the level of active hydrogenase in cells grown at either 30°C or 39°C in standard medium (MM) with or without the addition of both Fe (17 µM) and Ni (2.5 µM). The data showed that, together, the overexpression of the *hoxEFUYH* operon and the increased Ni- and Fe-availabilities led to an eight-fold higher amount of active hydrogenase (Figure 2). Though significant the gain in active hydrogenase remained lower than what was expected from the strong accumulation of *hoxEFUYH* transcripts and HoxF and HoxH proteins, suggesting that other limiting factors exist such as the abundance of the hypABCDEF proteins involved in assembly of the pentameric HoxEFUYH hydrogenase enzyme complex [2], [9].

### Construction and analysis of a mutant for concomitant temperature-controlled overexpression of the *hoxEFUYH* operon and *hypABCDEF* genes in *Synechocystis*

**Figure 3:**
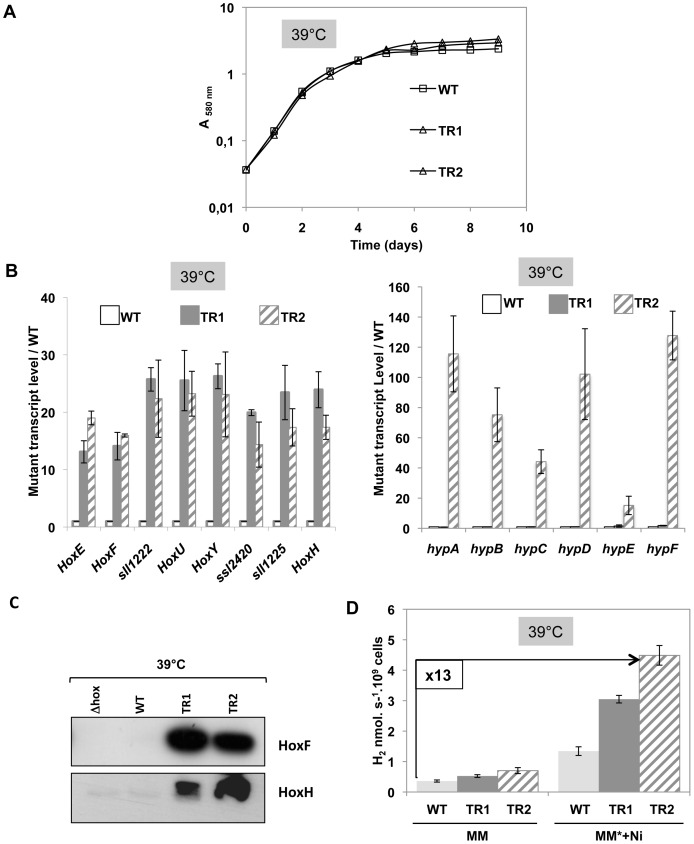
Analysis of the TR-*hoxEFUYH*-*hypABCDEF* mutant for temperature-regulated high-level expression of the *hoxEFUYH* and *hypABCDEF* genes. All experiments were performed at least three times on cells grown under standard light, at 39°C to induce the expression of the *hoxEFUYH* operon and the *hypABCDEF* genes controlled by the λ*c*I_857_-λ*p*_R_ system. (**A**) Typical growth of the WT (squares), TR-*hoxEFUYH* (TR1; white triangles) and TR-*hoxEFUYH*-*hypABCDEF* (TR2; grey triangles) strains. (**B**) Histogram plot representation of the transcript abundance (measured by Real-time quantitative PCR) of the eight genes of the *hoxEFUYH* operon (left part) and six *hypABCDEF* genes (right part) in WT (white bars), TR1 (grey bars) or TR2 (hatched bars) cells. (**C)** Western blot analysis of the abundance of the HoxF and HoxH proteins in WT, TR1 or TR2 cells. (**D**) Histograms representation of the hydrogenase activities of WT (light grey), TR1 (dark grey) or TR2 (hatched bars) growing in standard medium (MM) or MM* (MM+17 µM Fe) supplemented with 2.5 µM NiSO_4_.

To increase the formation of active hydrogenase enzymes, we decided to construct a mutant for temperature-controlled high-level expression of not only the *hoxEFUYH* operon but also all six genes *hypABCDEF* within the same cells (Figure 1). Therefore, the scattered *Synechocystis hypABCDEF* genes (Figure S1) were cloned in that order in our replicative plasmid vector pFC1 [5], under the control of the above mentioned λ*c*I_857_-λ*_PR_* system for tight temperature-controlled expression (Figure S8). First, the *hypA1* and *hypB1* genes were PCR amplified from *Synechocystis* DNA (see Table S2 for the primers), and joined through standard PCR-driven overlap extension [24] in a single DNA molecule flanked by a *Nde*I restriction site encompassing the ATG start codon of *hypA1* and a *Sal*I site downstream of the *hypB1* stop codon. After cleavage with both *Nde*I and *Sal*I, the *hypA1-hypB1* cassette was cloned in pFC1 opened with the same enzymes, yielding the pTR-*hypAB* plasmid. Then, the *hypC*, *hypD* and *hypE* genes were PCR amplified from *Synechocystis* DNA, joined in that order in a single DNA molecule, and cloned as a *Sal*I and *Bspe*I restriction DNA fragment in pTR-*hypAB* opened with the same enzymes. The resulting plasmid pTR-*hypABCDE* was opened with *Bsp*EI to clone the *Bsp*EI restriction fragment harboring the *hypF* gene generated by PCR, yielding the plasmid pTR-*hypABCDEF* (Figure S8). The structure of the pTR-*hypABCDEF* plasmid was verified by PCR analyses (Figure S9) and DNA sequencing of the l*c*I_857_-l*_PR_* cassette and all cloning junctions. Then pTR-*hypABCDEF* was introduced by conjugation [5] into the above-mentioned TR-*hoxEFUYH* mutant over-expressing the chromosomal *hoxEFUYH* operon in a temperature-regulated way. This yielded the Km^r^-Sm^r^/Sp^r^ mutant hereafter referred to as TR-*hoxEFUYH*-*hypABCDEF*, which also carried the WT alleles of the *hypABCDEF* genes in its chromosome (Figure 1). The TR-*hoxEFUYH*-*hypABCDEF* cells (abbreviated as TR2) grew as fit as the wild-type strain (WT) at 30°C or 39°C (Figure 3 and Figure S10). Then, we verified through quantitative RT-PCR that this mutant strongly expressed the *hoxEFUYH* and *hypABCDEF* genes in a temperature-controlled way, thanks to the λ*c*I_857_ and λ*p_R_* devices. Indeed, the abundance of the *hoxEFUYH* and *hypABCDEF* transcripts was much higher in TR-*hoxEFUYH*-*hypABCDEF* cells grown at 39°C than at 30°C, or as compared to WT cells grown at 30°C or 39°C. Consistently, the HoxF and HoxH proteins were much more abundant in TR-*hoxEFUYH*-*hypABCDEF* cells grown at 39°C, than at 30°C, or as compared to WT cells grown at either 30°C or 39°C (Figure 3). Furthermore, the level of active hydrogenase of the TR-*hoxEFUYH*-*hypABCDEF* mutant was higher in cells cultivated at 39°C than at 30°C, or as compared to WT cells grown at either 30°C or 39°C (Figure S10). Moreover, the gain of active hydrogenase observed at 39°C was significantly higher in the TR-*hoxEFUYH*-*hypABCDEF* mutant, which overexpresses the *hoxEFUYH* operon and the *hypABCDEF* genes, than in the TR-*hoxEFUYH* mutant, which overexpresses only the *hoxEFUYH* operon (Figure 3). This finding is consistent with the role of the HypABCDEF in assembling a functional HoxEFUYH hydrogenase complex [2], [9]. Collectively, our data demonstrated that the strong, temperature-controlled, expression of *hoxEFUYH* operon and the *hypABCDEF* genes are not detrimental to cell life. Again, the 13-fold increase in the amount of active hydrogenase observed in these TR-*hoxEFUYH*-*hypABCDEF* cells cultivated at 39°C, in the presence of additional Fe and Ni that positively influence hydrogenase activity, was low in comparison to the strong accumulation of the *hoxEFUYH* transcripts or HoxF and HoxH proteins (Figure 3 and Figure S10).

### Construction and analysis of a mutant for strong constitutive expression of the *hoxEFUYH* operon and *hypABCDEF* genes in *Synechocystis*

**Figure 4:**
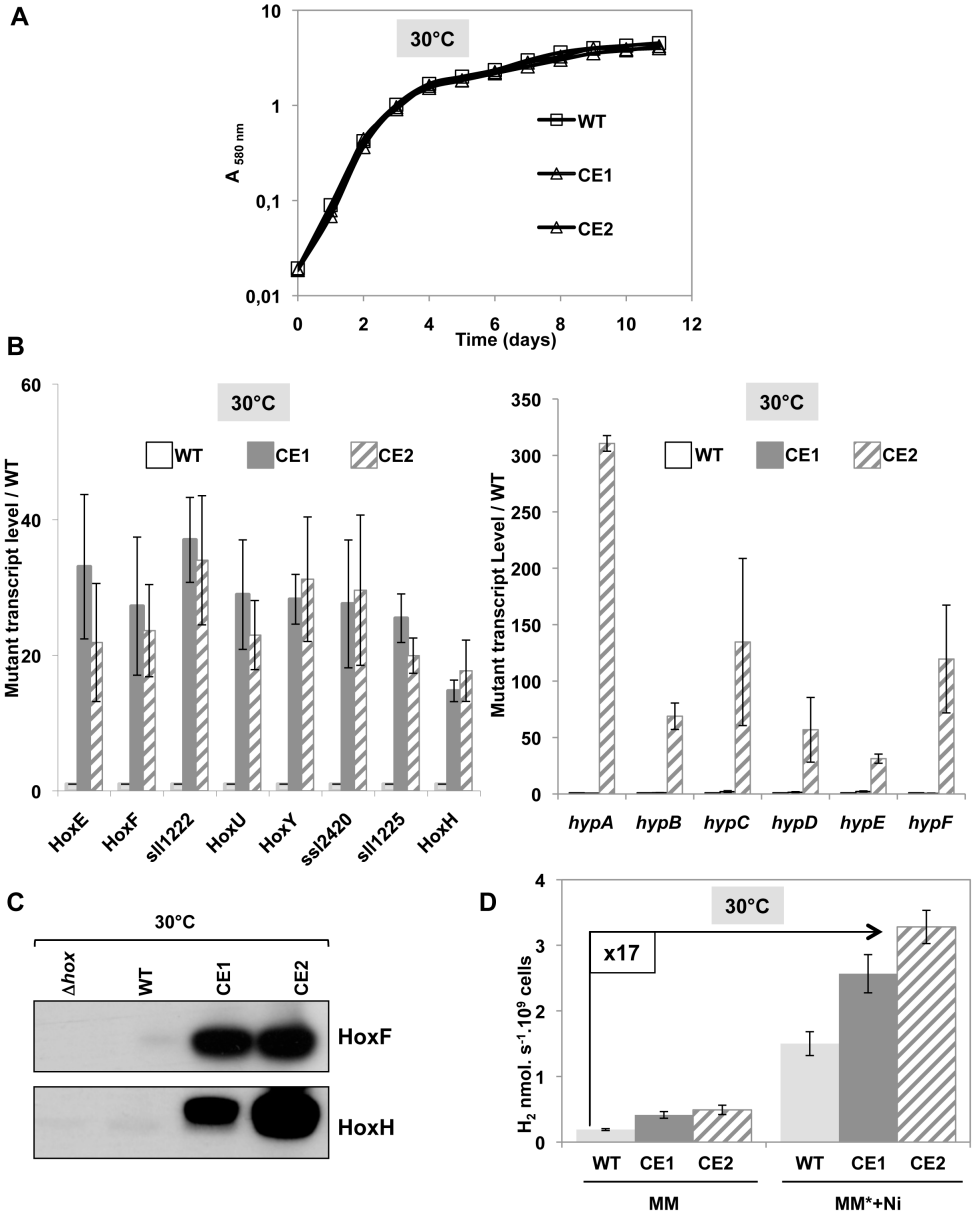
Analysis of the CE-*hoxEFUYH* and CE-*hoxEFUYH*-*hypABCDEF* mutants for strong constitutive expression of the *hoxEFUYH* alone or together with the *hypABCDEF* genes. All experiments were performed at least three times on cells grown at 30°C under standard light. (**A**) Typical growth of the WT (squares), CE-*hoxEFUYH* (CE1; white triangles) and CE-*hoxEFUYH*-*hypABCDEF* (CE2; grey triangles) cells. (**B**) Histogram plot representation of the transcript abundance (measured by Real-time quantitative PCR) of the eight genes of the *hoxEFUYH* operon (left part) and the six *hypABCDEF* genes (right part) in the CE1 (grey bars) or CE2 (hatched bars) mutants, as compared to the WT strain (white bars). (**C**) Western blot analysis of the abundance of the HoxF and HoxH proteins in WT, CE1 or CE2 cells. (**D**) Histograms representation of the hydrogenase activities of WT (light grey), CE1 (dark grey) or CE2 cells (hatched bars) growing in standard medium (MM) or MM* (MM+17 µM Fe) supplemented with 2.5 µM NiSO_4_.

The present finding that the concomitant high-level expression of the *hoxEFUYH* and *hypABCDEF* genes was not toxic to the temperature-controlled mutant (TR-*hoxEFUYH*-*hypABCDEF*) growing at 39°C, prompted us to attempt further increasing it in a constitutive way as a step towards the engineering of a powerful strain for hydrogen production as well as to facilitate the comparative analysis of such a mutant with the WT and our D*hoxEFUYH* mutant (Δ*hoxEFUYH*::Km^r^). Therefore, the plasmids pTR-*hoxEFUYH* and pTR-*hypABCDEF* were deleted of most of the λ*c*I_857_ gene encoding the temperature-sensitive repressor because it is not totally inactivated at 39°C [5] and *Synechocystis* grows poorly at higher temperatures. Practically, the pTR-*hoxEFUYH* and pTR-*hypABCDEF* plasmids were cleaved with the restriction enzyme *Psi*I to delete (517 bp) the λ*c*I_857_ repressor gene yielding the pCE-*hoxEFUYH* and pCE-*hypABCDEF* plasmids (CE for constitutive expression). The pCE-*hoxEFUYH* plasmid (Figure S11) was linearized with *Eco*RV and transformed to *Synechocystis*, where homologous recombinations introduced the Km^r^-λ*p_R_* DNA cassette in place of the weak [10] *hoxEFUYH* operon promoter, in all copies of the polyploïd [19] chromosome (Figure S12). This Km^r^ CE-*hoxEFUYH* mutant grew as fit as the WT strain in standard (30°C) photoautotrophic conditions (Figure 4). Then, we verified through quantitative RT-PCR that this mutant strongly expressed all eight genes of the *hoxEFUYH* operon, thanks to the λ*p_R_* promoter, and accumulated the HoxF and HoxH proteins (Figure 4). Consistently, the level of active hydrogenase in the CE-*hoxEFUYH* mutant was higher than that of the WT strain (Figure 4). Together, the constitutive overexpression of the *hoxEFUYH* operon and the increased Ni- and Fe-availabilities led to a fourteenth-fold higher hydrogenase activity (Figure 4), i.e. a higher increase than the eight-fold value observed after heat induction of the temperature-controlled TR-*hoxEFUYH* mutant (Figure 2).

To increase the formation of active hydrogenase enzymes, we introduced the above-mentioned pCE-*hypABCDEF* plasmid (Sm^r^/Sp^r^; Figure S13) by conjugation [5] into the CE-*hoxEFUYH* mutant. This yielded the Km^r^-Sm^r^/Sp^r^ mutant hereafter referred to as CE-*hoxEFUYH*-*hypABCDEF*, which also carried the WT alleles of the *hypABCDEF* genes in its chromosome (Figure 1). This CE-*hoxEFUYH*-*hypABCDEF* mutant grew as fit as the WT strain and the CE-*hoxEFUYH* mutant in standard (30°C) photoautotrophic conditions (Figure 4). As expected, the CE-*hoxEFUYH*-*hypABCDEF* mutant strongly expressed the *hoxEFUYH* operon and the *hypABCDEF* genes accumulated the corresponding proteins (Figure 4; and Table S3). Furthermore, the level of active hydrogenase of the CE-*hoxEFUYH*-*hypABCDEF* mutant was higher than those of the CE-*hoxEFUYH* mutant and the WT strain in that order (Figure 4). Together, the constitutive overexpression of the *hoxEFUYH* operon and the *hypABCDEF* genes, and the increased Ni- and Fe-availabilities led to a seventeenth-fold increase in the level of active hydrogenase as compared to WT cells cultivated in absence of Fe and Ni supplementation (Figure 4). We think this strain with an increased amount of active hydrogenase is a suitable chassis for future gene manipulations required to engineer a powerful hydrogen producer.

### The *hoxEFUYH* operon, but not the *hypABCDEF* genes, operates in the protection against hydrogen peroxide

**Figure 5:**
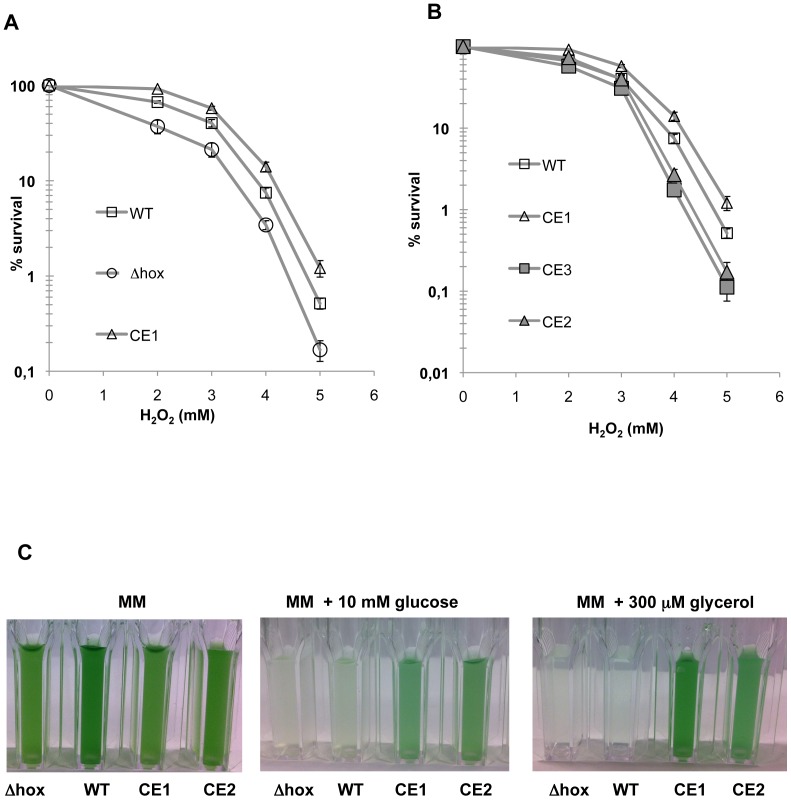
Influence of the *hoxEFUYH* and *hypABCDEF* genes on the tolerance to H_2_O_2_, glucose or glycerol. (**A**) Typical survival of the *Synechocystis* strains WT (open squares), Δ*hoxEFUYH*::Km^r^ (Δhox, open cercles) or CE-*hoxEFUYH* (CE1, open triangles) grown at 30°C and challenged for 1 hour with H_2_O_2_ under anaerobiose before plating under standard photoautotrophic conditions. (**B**) Typical survival to the anaerobic H_2_O_2_ stress of the WT strain (open squares), CE1 (open triangles), CE-*hoxEFUYH*-*hypABCDEF* (CE2, dark-grey triangles), CE-*hypABCDEF* (CE3, dark-grey squares). (**C**) Typical photoautotrophic growth of the wild-type (WT), D*hoxEFUYH*::Km^r^ (Δhox), CE-*hoxEFUYH* (CE1) and CE-*hoxEFUYH*-*hypABCDEF* (CE2) strains. Cells grown twice in standard (MM) liquid medium (up to mid-log phase) were inoculated in MM without or with glucose 10 mM or 300 µM glycerol, and incubated under light (3,000 luxes) during 7 days. All experiments were performed at least three times.

All aerobic organisms invariably produce reactive oxygen species, such as O_2_^-^ (superoxide anion) and H_2_O_2_ through the accidental autoxidation of redox enzymes [25], which occurs when their reduced cofactors accidentally reduces oxygen. This phenomenon is frequent in cyanobacteria, where their active photosynthesis massively produces oxygen and often generates an excess of electrons [26]. As the cyanobacterial hydrogenase enzyme complex has been proposed to act as an electron valve releasing some of the supernumerary electrons [9], we have tested the H_2_O_2_ tolerance of the presently reported hydrogenase mutants under anaerobic conditions. The Δ*hoxEFUYH*::Km^r^ (deletion) mutant appeared to be more sensitive to H_2_O_2_ than the WT strain and the CE-*hoxEFUYH* mutant in that order (Figure 5). Similar, but smaller, differences in the H_2_O_2_ tolerance of the various strains were observed under standard photoautotrophic (aerobic) conditions (data not shown). These findings support the proposal that the hydrogenase enzyme act as an electron valve without ruling the possibility that the Hox enzyme also participates in the detoxification of H_2_O_2_. By contrast, the overexpression of the *hypABCDEF* genes actually decreased the tolerance to H_2_O_2_, as shown by the comparison of the WT strain and the CE-*hypABCDEF* mutant on one hand, and the mutants CE-*hoxEFUYH* and CE-*hoxEFUYH*-*hypABCDEF* on the other hand (Figure 5). Collectively, these findings showed that the *hoxEFUYH* operon and the *hypABCDEF* genes contribute to H_2_O_2_ tolerance, positively (*hoxEFUYH* operon) and negatively (*hypABCDEF*). Future experiments will be required to test whether the higher H_2_O_2_ tolerance directed by the overexpression of the *hoxEFUYH* operon is due, directly or indirectly, to the increased abundance of (i) the HoxHY hydrogenase enzyme *per se*, (ii) the HoxEFU diaphorase enzyme, and/or (iii) the Sll1222, Ssl2420 and Sll1225 proteins of as yet unknown function.

### The *hoxEFUYH* operon operates in the defense against the stress (likely redox) triggered by the reduced carbon metabolites glucose and glycerol

Several lines of previous evidences prompted us to test the influence of the reduced carbon metabolites glycerol and glucose on the aerobic growth of the hydrogenase mutants presently reported. Glycerol, a cheap surplus of saponification and biodiesel industries [27], and glucose were both shown to stimulate hydrogen photoproduction in the cyanobacteria *Cyanothece* ATCC 51142 [28] and *Arthrospira* (*Spirulina*) *maxima* CS-328 [23], respectively. These data suggested that hydrogen photoproduction is often limited by reductant availability. In addition, we have previously reported that glucose is toxic to *Synechocystis* incubated under an otherwise physiological illumination [29], likely because it decreases CO_2_-assimilation that normally consume a large number of photosynthetic electrons, thereby enabling the spared electrons to recombine with O_2_ to generate toxic reactive oxygen species. Like glucose, glycerol was found to be toxic to the WT strain growing aerobically under a normal light fluence (Figure 5), in agreement with a previous report [30]. In contrast, the presently constructed hydrogenase overproducing mutants CE-*hoxEFUYH* and CE-*hoxEFUYH*-*hypABCDEF* were killed by neither glucose nor glycerol (Figure 5). These findings are consistent with the proposal that the Hox hydrogenase enzyme reoxidizes NAD(P)H during fermentative conditions to allow catabolism of endogenous carbohydrates to proceed [9]. Collectively these findings strengthened the proposal that hydrogenase operates as an electron valve, preventing supernumerary photosynthetic electrons to recombine with O_2_ to generate toxic reactive oxygen species.

## Discussion

In spite of valuable previous studies, the role of the hydrogen metabolism remains puzzling in cyanobacteria, and the activity of the Ni-Fe hydrogenase, the hydrogen-producing enzyme, remains low [2], [8], [9]. Therefore, we have studied the hydrogen production machine of the model unicellular strain *Synechocystis* PCC6803. This was done through deletion of the hydrogenase-encoding *hoxEFUYH* operon, as well as the overexpression of the *hoxEFUYH* operon alone, or in combination with the six *hypABCDEF* genes (Figure 1) which operate in hydrogenase assembly and are scattered in the *Synechocystis* genome (Figure S1). The Δ*hoxEFUYH*::Km^r^ deletion mutant (Figures S2–4) grew as fit as the wild-type (WT) strain (Figure S5), showing that the *hoxEFUYH* operon is dispensable to *Synechocystis*. This finding is consistent with the dispensability of the *hoxHY* genes in the otherwise WT strain [20], and of the *hoxEFUYH* operon in the glucose tolerant mutant [2]. To increase the expression of the *hoxEFUYH* operon and the *hypABCDEF* genes, while caring with the possibility that above a certain level they might become toxic, we used the λ*c*I_857_-λ*p_R_* system that expresses genes proportionally to the temperature of growth [4], [5], [21]: *i.e.* absence of expression at 30°C (the standard growth temperature) and strong expression at 39°C (good growth of wild-type cells). Practically (Figures S6–10), we replaced the weak [10] natural promoter of the *hoxEFUYH* operon by the Km^r^-λ*c*I_857_-λ*p_R_* DNA cassette, and we cloned the *hypABCDEF* genes under the control of the same λ*c*I_857_-λ*p_R_* system, in the Sm^r^/Sp^r^ plasmid pFC1, which replicates at the same 10–20 copies per cell as the chromosome [5]. As expected, the resulting mutants expressed the *hoxEFUYH* operon alone (TR-*hoxEFUYH* mutant) or together with the *hypABCDEF* genes (TR-*hoxEFUYH*-*hypABCDEF* mutant) in a temperature-controlled way (no expression at 30°C, strong expression at 39°C; Figures 2–3), and the 39°C-induced strong expression of the *hoxEFUYH* operon and the *hypABCDEF* genes was not detrimental to cell fitness. In the presence of higher Ni- and Fe-availabilities, the overexpression of the *hoxEFUYH* operon alone or in combination with the *hypABCDEF* genes led to eight-fold or thirteen-fold increased amounts of active hydrogenase, respectively (Figure 2). To our knowledge the TR-*hoxEFUYH*-*hypABCDEF* mutant is the first cyanobacterial strain capable of overexpressing simultaneously the endogenous *hoxEFUYH* and *hypABCDEF* genes *in vivo*. Thanks to the tight temperature-control of the hydrogenase activity of this mutant (none at 30°C, strong at 39°C) it will be possible in the future to use high-throughput “omics” techniques to perform a kinetic analysis of the global cell responses to hydrogen production, to attempt distinguishing between rapid (likely specific) responses, and slow (likely indirect) responses.

To further increase the production of active hydrogenase we deleted the λ*c*I_857_ repressor-encoding gene from the above-mentioned constructions, because it is not totally inactivated at 39°C [5] and *Synechocystis* grows poorly at higher temperatures. The resulting mutants CE-*hoxEFUYH* and CE-*hoxEFUYH*-*hypABCDEF* (CE for strong constitutive expression) grew as fit as WT cells (Figure 4) and exhibited higher levels of active hydrogenase than their temperature-regulated counterparts (fourteen-fold in CE-*hoxEFUYH* and seventeen-fold in CE-*hoxEFUYH*-*hypABCDEF* as compared to WT cells growing in standard medium). All mutants displayed higher levels of expression of the *hoxEFUYH* and *hypABCDEF* genes than that of active hydrogenase (Figure 4) indicating that other limiting factors should be dealt with. Therefore, we will pay a particular attention to glutathionylation (the formation of a mixed-disulfide between the cysteines residues of a protein and glutathione (the anti-oxidant tripeptide γ-glutamyl-cysteinyl-glycine) because we recently reported that the AbrB2 hydrogen regulator, and the mercuric reductase enzyme, can be glutathionylated [15], [21].

Meanwhile, the presently constructed mutants proved useful to advance our understanding on the physiological role of the hydrogenase enzyme, which was very limited so far. We compared the H_2_O_2_ tolerance of the WT and mutant strains to test whether the cyanobacterial hydrogenase enzyme truly acts as an electron valve releasing excess of photosynthetic electrons [9] to prevent their recombination with O_2_ that generates toxic reactive oxygen species (ROS). We found that the *hoxEFUYH* operon and the *hypABCDEF* genes contribute to the protection against H_2_O_2_ (Figure 5), positively (*hoxEFUYH* operon) and negatively (*hypABCDEF*). In addition, we tested the influence on our mutants of the reduced-carbon metabolites glucose and glycerol, a cheap surplus of industries [27], because they stimulated hydrogen production in the cyanobacteria *Arthrospira* (*Spirulina*) *maxima* [23] and *Cyanothece* ATCC 51142 [28]. Both glucose and glycerol were toxic to *Synechocystis* growing under an otherwise normal light fluence (Figure 5), probably because these reduced metabolites somehow decline the electrons-consuming CO_2_-assimilation, thereby allowing spared electrons to recombine with O_2_ and generate ROS. By contrast, neither glucose nor glycerol killed the hydrogenase overproducing mutants CE-*hoxEFUYH* and CE-*hoxEFUYH*-*hypABCDEF* (Figure 5), in agreement with the proposal that the Hox hydrogenase enzyme reoxidizes NAD(P)H that serves for the catabolism of carbohydrates [9]. Collectively our findings strengthened the proposal that hydrogenase operates as an electron valve, preventing supernumerary photosynthetic electrons to recombine with O_2_ to generate toxic ROS. We view the hydrogenase complex as an important enzyme to cyanobacteria as *Synechocystis*, which likes growing as biofilm, a thick network of autoaggregated cells [31], where the cells are certainly frequently exposed to H_2_O_2_ and reduced metabolites released by their neighbours (living, or dying and lysing). This view is conforted by the absence of hydrogenase enzyme in most planktonic cyanobacteria living in open oceans.

## Conclusions

Using gene deletion and overexpression we have shown that the *hoxEFUYH* operon operates in the defence against H_2_O_2_, glycerol and glucose stresses, and that the simultaneous overproduction of the HoxEFUYH and HypABCDEF proteins led to a 20-fold increase in active hydrogenase. We think that our sophisticated mutants with higher hydrogenase contents and a healthy growth will be very useful for the purification of large hydrogenase quantities for structural analyses; to continue deciphering the biological role of the hydrogen production machine; and for further genetic manipulations aiming at increasing the O_2_ tolerance of the hydrogenase enzyme for a better hydrogen production.

## Supporting Information

Figure S1 **Schematic representation of hydrogen production machine in** ***Synechocystis*** **PCC6803 adapted from** [**9**] **.** The genes are represented by arrows, which point in the direction of their transcription (http://bacteria.kazusa.or.jp/cyanobase/), and are colored similarly to their protein products. The green numbers indicate the spacing distance (in kilobases) between the scattered genes. The *hoxEFUHY* operon is weakly transcribed [10] as the polycistronic mRNA (bent blue arrow), which encodes (i) the hydrogenase sub-complex (made by the HoxY protein and the HoxW-matured HoxH subunit); (ii) the HoxEFU diaphorase sub-complex; and (iii) the three proteins of unknown function (white forms). The electron transfer FMN cofactor, Fe-Ni center, and [4Fe-4S] and [2Fe-2S] clusters of the Hox proteins, are represented by the blue squares, the pink star, dark-red squares and light-red diamonds, respectively. The zinc-bound to HypA1 and HypB1 proteins is shown as the blue form. CP designates carbamoyl phosphate. The brown lines stand for the reversible inactivation of Hox activity mediated by oxygen. The photosynthetic membrane is represented in green.
(TIFF)

Figure S2 **Construction of the Km^r^ pFC1K plasmid for temperature regulated gene expression in** ***Synechocystis*** **.** The genes are represented by large arrows, which point in the direction of their transcription. The red triangle indicates the strong λ*p*_R_ promoter followed by the λ*cro* ribosome-binding site (5′-AGGA-3′) and ATG start codon embedded within a unique *Nde*I restriction site (5′-CATATG-3′) for in frame-fusion of the studied protein-coding regions. They are expressed in a temperature-controlled way thanks to the λ*c*I_857_ gene (hatched arrow), which encodes the temperature-sensitive repressor that tightly controls λ*p*_R_. The transcription and translation stop signals (TT) preventing read-through of gene expression from the antibiotic resistance gene (Sp^r^/Sm^r^ in pFC1 or Km^r^ in pFC1K) are indicated by the black bars.
(TIFF)

Figure S3 **Construction of the Δ** ***hoxEFUYH*** **::Km^r^ DNA cassette for the deletion of the** ***Synechocystis hoxEFUYH*** **operon, which comprises the** ***hoxE*** **,** ***hoxF*** **,** ***sll1222*** **,** ***hoxU*** **,** ***hoxY*** **,** ***ssl12420*** **,** ***sll1225*** **and** ***hoxH*** **genes in that order (** **Figure 1** **and Figure S1).** The genes are represented by white (sll1222, ssl2420 and sll1225) or pink (*hoxE*, *hoxF*, *hoxU*, *hoxY* and *hoxH*) boxes, which point in the direction of their transcription. The transcription terminator (TT) preventing the read-through of expression from the Km^r^ gene is represented by the vertical grey rectangle. The rectangles designated as hoxup2 and hoxdwn represent the DNA regions flanking the *hoxEFUYH* operon, which served as platforms for homologous recombinations promoting the targeted replacement of the *hoxEFUYH* operon by the Km^r^ gene.
(TIFF)

Figure S4 **PCR verification of the Δ** ***hoxEFUYH*** **::Km^r^ mutant showing that the replacement of the** ***hoxEFUYH*** **operon by the Km^r^ marker occurred in all copies of the polyploïd chromosome of** ***Synechocystis*** **.** (**A**) Schematic representation of the *hoxEFUYH* operon locus in the wild-type strain (WT) and the Δ*hoxEFUYH*::Km^r^ mutant (Δhox), which harbors the Km^r^ marker in place of the whole *hoxEFUYH* operon (from 58 bp upstream of the *hoxE* ATG start codon, to 8 bp downstream of the *hoxH* TAA stop codon). The small colored triangles represent the oligonucleotides primers (Table S2) that generated the PCR DNA segments (double arrows) typical of the WT strain or the Δhox mutant. (**B**) UV-light image of the agarose gel showing the 7 kb and 2.4 kb PCR-1 products typical of the chromosome organization in the WT strain and the Dhox mutant growing in standard conditions. Marker (M_F_)  = 1 Kb plus DNA Ladder (Fermentas). (**C**) PCR-2 and (**D**) PCR-3 confirmation that Δhox mutant cells contain only Dhox mutant (no WT) chromosomes. Marker (M_I_)  = 1 Kb plus DNA Ladder (Invitrogen).
(TIFF)

Figure S5 **Analysis of the** ***Synechocystis*** **Δ** ***hoxEFUYH*** **::Km^r^ mutant (Δhox).** (**A**) Schematic representation of the *hoxEFUYH* operon locus in the WT strain or the Δhox mutant. (**B**) Typical growth of the WT (squares) and Δhox cells (circles) in standard conditions at either 30°C (open symbols) and 39°C (grey symbols). (**C**) Western blot analysis of the abundance of the HoxF and HoxH proteins in WT and Δhox cells grown at 30°C or 39°C. (**D**) Histograms representation of the hydrogenase activities of WT and Δhox cells grown at 30°C or 39°C. These experiment were performed three times.
(TIFF)

Figure S6 **Construction of the Km^r^-λ** ***c*** **I_857_-λ** ***p_R_*** **DNA cassette for temperature controlled expression of the** ***Synechocystis hoxEFUYH*** **operon.** The genes are represented by large arrows, which indicate the direction of their transcription. The strong **λ** *p*_R_ promoter is represented by the red triangle oppositely oriented to the l*c*I_857_ gene, which encodes the temperature-sensitive repressor that tightly controls **λ** *p*_R_. The transcription and translation stop signals (TT), which prevent read-through of gene expression from the Km^r^ marker are indicated by the vertical grey bar. The hoxup region of DNA (purple rectangle) upstream of the *hoxEFUYH* operon promoter and the *hoxE* gene served as platform for homolous recombinations, which introduced the Km^r^-**λ** *c*I_857_-**λ** *p_R_* DNA cassette in place of the weak promoter [10] of the *hoxEFUYH* operon.
(TIFF)

Figure S7 **PCR verification of the** ***Synechocystis*** **TR-** ***hoxEFUYH*** **mutant (TR1) for temperature regulated high-level expression of the** ***hoxEFUYH*** **operon.** (**A**) Schematic representation of the *hoxEFUYH* operon locus in the WT strain or the TR1 mutant, which harbors the Km^r^-**λ** *c*I_857_-**λ** *p_R_* cassette in place of the natural 691 bp-long *hoxEFUYH* promoter region (starting from the first bp upstream of the *hoxE* ATG start codon). The oligonucleotides primers represented by small colored triangles (Table S2) served for the PCR verifications indicated by double arrows. (**B**) UV-light image of the agarose gel showing the 1.5 kb and 3.6 kb DNA products of the PCR-1 analysis of the genome of the WT strain or the TR1 mutant. Marker (M_I_)  = 1 Kb plus DNA Ladder (Invitrogen). (**C**) PCR-2 and (**D**) PCR-3 confirmation that TR1 mutant cells contain only TR1 mutant (no WT) chromosomes. Marker (M_B_)  = 1 Kb plus DNA Ladder (Biolabs).
(TIFF)

Figure S8 **Construction of the pTR-** ***hypABCDEF*** **plasmid for temperature regulated expression of the** ***Synechocystis hypABCDEF*** **genes.** For the sake of clarity, the four genes *hypB1* (sll1432), *hypC* (ssl3580), *hypE* (sll1462) and *hypF* (sll0322) are represented oppositely to their natural orientation (Figure 1 and Figure S1). The small colored arrows indicate the position of the oligonucleotide primers used for the PCR amplification (dashed lines) and assembly (blue arrows) used for cloning the *hypABCDEF* genes into the pFC1 vector [5], yielding pTR-*hypABCDEF*. These PCR primers (Table S2) are namely: HypA1NdeIFwd (blue rightward-pointing arrow) and HypA1ASSRv (yellow leftward-pointing arrow) for PCR1; HypB1ASSFwd (yellow rightward-pointing arrow) and HypB1SalIRv (green leftward-pointing arrow) for PCR2; HypCSalIfwdbis (green rightward-pointing arrow) and HypCASSrvbis (orange leftward-pointing arrow) for PCR3; HypEASSfwd (purple rightward-pointing arrow) and HypEBspeIrv (red leftward-pointing arrow) for PCR4; HypDASSfwd (orange rightward-pointing arrow) and HypDASSrv (purple leftward-pointing arrow) for PCR5; and HypFBspeIfwdbis (red rightward-pointing arrow) and HypFBspeIrv (red leftward-pointing arrow) for PCR6. The **λ** *p*_R_ promoter is represented by the red triangle oppositely oriented to the l*c*I_857_ repressor-encoding gene. The transcription and translation stop signals (TT) preventing read-through of gene expression are indicated by grey bars.
(TIFF)

Figure S9 **PCR verification of the pTR-** ***hypABCDEF*** **plasmid.** (**A**) Schematic representation of the *hypABCDEF* genes (grey boxes) in the pTR-*hypABCDEF* plasmid replicating in *E. coli* (lane C+ for positive control) or in the *Synechocystis* mutant designated as TR-*hoxEFUYH*-*hypABCDEF* (TR2). The oligonucleotides primers (Table S2) used to generate the pTR-*hypABCDEF* specific DNA segments (dashed lines) of the following sizes: 1.3 kb (PCR1, panel **B**); 2.6 kb (PCR2, panel **C**) and 770 bp (PCR3, panel **D**) are namely: HypA1NdeIFwd (blue arrow) and HypB1SalIRv (green leftward-pointing arrow) for PCR1; HypCSalIfwdbis (green rightward-pointing arrow) and HypFBspeIFwdBis (red arrow) for PCR2; and HypDASSrv (purple leftward-pointing arrow) and HypEASSfwd (purple rightward-pointing arrow) for PCR3. Marker (M_F_)  = 1 Kb plus DNA Ladder (Fermentas). Note that the PCR1-3 reactions can amplify only the adjacent *hypABCDEF* genes present in the pTR-*hypABCDEF* plasmid, not the chromosomal *hypABCDEF* genes because they are located too far away from each others (see Figure S1 and Figure S8). This explains the absence of PCR products in the negative-control *Synechocystis* strain TR1 (the TR-*hoxEFUYH* mutant), which lacks pTR-*hypABCDEF*.
(TIF)

Figure S10 **Confirmation of the temperature-controlled high-level expression of the** ***hoxEFUYH*** **operon and the** ***hypABCDEF*** **genes in the** ***Synechocystis*** **mutant TR-** ***hoxEFUYH*** **-** ***hypABCDEF*** **.** All experiments were performed at least three times on cells grown under standard light at 30°C or 39°C. (**A**) Typical growth of the WT (squares) and TR-*hoxEFUYH*-*hypABCDEF* (TR2; triangles) at 30°C (white symbols) or 39°C (grey symbols). (**B**) Histogram plot representation of the transcript abundance (measured by Real-time quantitative PCR) of the *hoxEFUYH* operon (left part) and the *hypABCDEF* genes (right part) in WT (white bars) or TR2 (hatched bars) cells. (**C**) Western blot analysis of the abundance of the HoxF and HoxH proteins in WT or TR2 cells. (**D**) Histograms representation of the hydrogenase activities of WT (light grey), or TR2 (hatched bars) growing in standard medium (MM) or MM* (MM + 17 µM Fe) supplemented with 2.5 µM NiSO_4_.
(TIFF)

Figure S11 **Construction of the Km^r^-λ** ***p_R_*** **DNA cassette for constitutive strong expression of the** ***Synechocystis hoxEFUYH*** **operon.** The genes are represented by large arrows, while the dark bar indicates the transcription and translation stop signals (TT), which prevent read-through of gene expression from the Km^r^ marker. The strong **λ** *p*_R_ promoter is represented by the red triangle oppositely oriented to the repressor encoding-gene **λ** *c*I_857_, which was inactivated by *Psi*I restriction during the construction of the Km^r^-**λ** *p_R_* DNA cassette. The 252 bp hoxup region of DNA upstream of the *hoxEFUYH* operon promoter and the *hoxE* gene, served as platform for homologous recombinations that introduced the Km^r^-**λ** *p_R_* DNA cassette in place of the weak natural promoter of the *hoxEFUYH* operon.
(TIFF)

Figure S12 **PCR verification of the CE-** ***hoxEFUYH*** **mutant for strong constitutive expression of the** ***hoxEFUYH*** **operon.** (**A**) Schematic representation of the *hoxEFUYH* operon in the WT strain or the CE-*hoxEFUYH* mutant (CE1), which harbors the Km^r^-**λ** *p_R_* DNA cassette in place of the natural 691 bp-long *hoxEFUYH* promoter region (starting from the first bp upstream of the *hoxE* ATG start codon). The oligonucleotides primers represented by small colored triangles (Table S2) served for the PCR verifications indicated by double arrows. (**B**) UV-light image of the agarose gel showing the 1.5 kb and 3.0 kb DNA products of the PCR-1 analysis of the WT strain or the CE1 mutant. Marker (M_I_)  = 1 Kb plus DNA Ladder (Invitrogen). (**C**) PCR-2 and (**D**) PCR-3 confirmation that CE1 mutant cells contain only CE1 mutant (no WT) chromosomes. Marker (M_B_)  = 1 Kb plus DNA Ladder (Biolabs).
(TIFF)

Figure S13 (**A**) Construction of the pCE-*hypABCDEF* plasmid for constitutive strong expression of the *hypABCDEF* genes. pCE-*hypABCDEF* was generated after the *Psi*I cleavage and religation of the pTR-*hypABCDEF* plasmid to inactivate the λ*c*I_857_ repressor gene, which normally controls the strong λ*p*_R_ promoter (red triangle). The genes are represented by large arrows while the transcription and translation stop signals (TT) are indicated by dark grey bars. (**B**) Schematic representation of the *hypABCDEF* genes in the pCE-*hypABCDEF* plasmid replicating in *E. coli* (lane C+ for positive control) or in the *Synechocystis* mutant designated as CE-*hoxEFUYH*-*hypABCDEF* (CE2). The oligonucleotides primers (Table S2) used to generate the pCE-*hypABCDEF* specific DNA segments (dashed lines) of the following sizes: 1.3 kb (PCR1, panel **B**); 2.6 kb (PCR2, panel **C**) and 770 bp (PCR3, panel **D**) are namely: HypA1NdeIFwd (blue arrow) and HypB1SalIRv (green leftward-pointing arrow) for PCR1; HypCSalIfwdbis (green rightward-pointing arrow) and HypFBspeIfwdbis (red arrow) for PCR2; and HypDASSrv (purple leftward-pointing arrow) and HypEASSRwd (purple rightward-pointing arrow) for PCR3. Marker (M_F_)  = 1 Kb plus DNA Ladder (Fermentas). Note that the PCR1-3 reactions can amplify only the adjacent *hypABCDEF* genes present in the pCE-*hypABCDEF* plasmid, not the chromosomal *hypABCDEF* genes because they are located too far away from each others (see Figure S1 and Figure S8). This explains the absence of PCR products in the negative-control *Synechocystis* strain CE1 (the CE-*hoxEFUYH* mutant), which lacks pCE-*hypABCDEF*.
(TIF)

Table S1 **Characteristics of the plasmids and strains used in this study.**
(DOCX)

Table S2 **Oligonucleotide primers used in this study.**
(DOCX)

Table S3 **List of the Hox and Hyp hydrogenase proteins detected in Synechocystis WT strain or CE2 mutant grow in standard conditions using LC-MS/MS (Orbitrap) or LCMS/MS(Q-Exactive) techniques. ND: Non Detected.**
(DOCX)
