# Supplementary figures and images for: Engineering Synechocystis PCC6803 for Hydrogen Production: Influence on the Tolerance to Oxidative and Sugar Stresses
Source: PLoS One. 2014 Feb 24;9(2):e89372. doi: 10.1371/journal.pone.0089372 (PMC3933540; doi:10.1371/journal.pone.0089372)

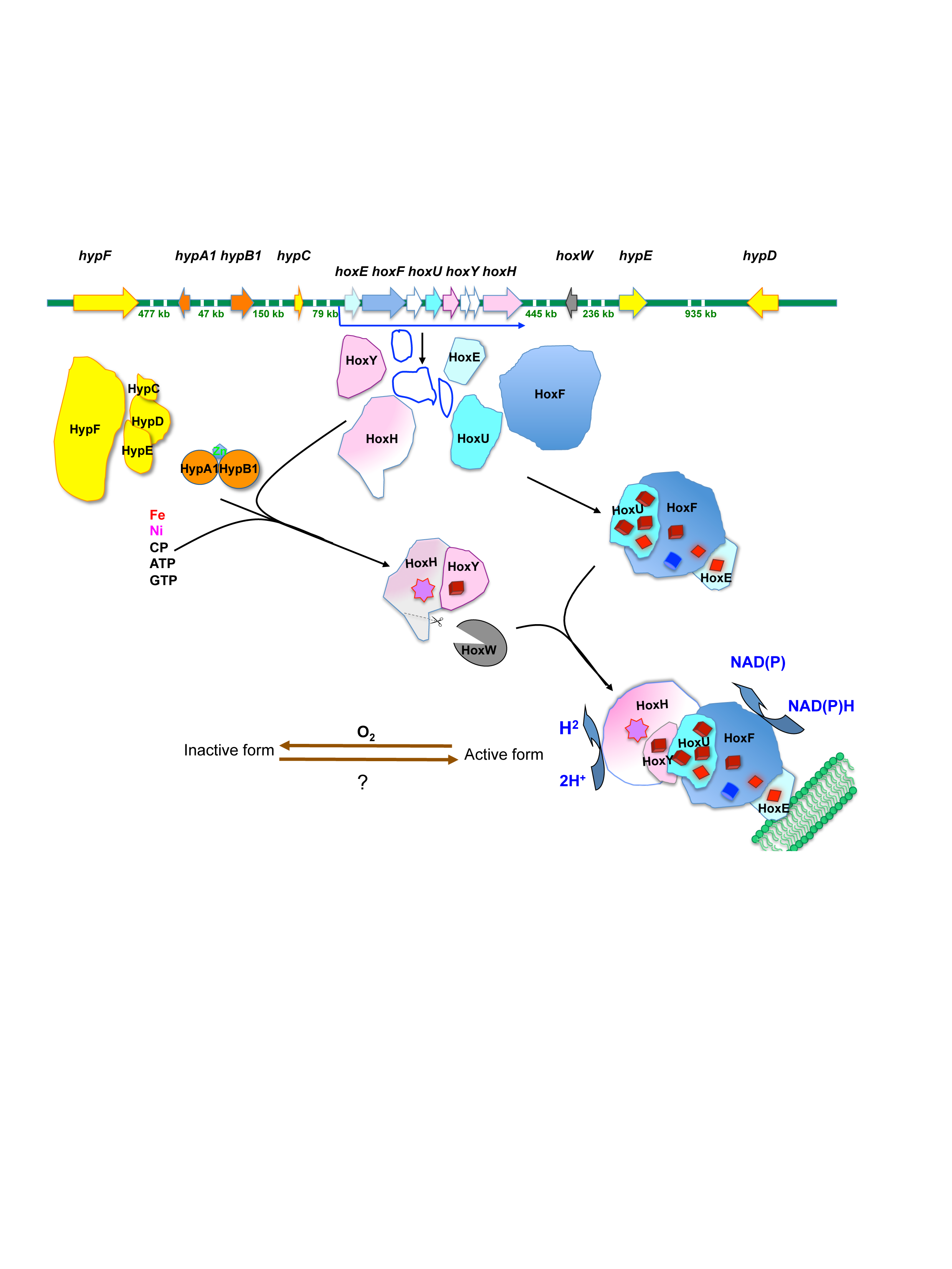

Supplement: Figure S1 — Schematic representation of hydrogen production machine in Synechocystis PCC6803 adapted from [9] . The genes are represented by arrows, which point in the direction of their transcription (http://bacteria.kazusa.or.jp/cyanobase/), and are colored similarly to their protein products. The green numbers indicate the spacing distance (in kilobases) between the scattered genes. The hoxEFUHY operon is weakly transcribed [10] as the polycistronic mRNA (bent blue arrow), which encodes (i) the hydrogenase sub-complex (made by the HoxY protein and the HoxW-matured HoxH subunit); (ii) the HoxEFU diaphorase sub-complex; and (iii) the three proteins of unknown function (white forms). The electron transfer FMN cofactor, Fe-Ni center, and [4Fe-4S] and [2Fe-2S] clusters of the Hox proteins, are represented by the blue squares, the pink star, dark-red squares and light-red diamonds, respectively. The zinc-bound to HypA1 and HypB1 proteins is shown as the blue form. CP designates carbamoyl phosphate. The brown lines stand for the reversible inactivation of Hox activity mediated by oxygen. The photosynthetic membrane is represented in green. (TIFF) [file pone.0089372.s001.tiff]

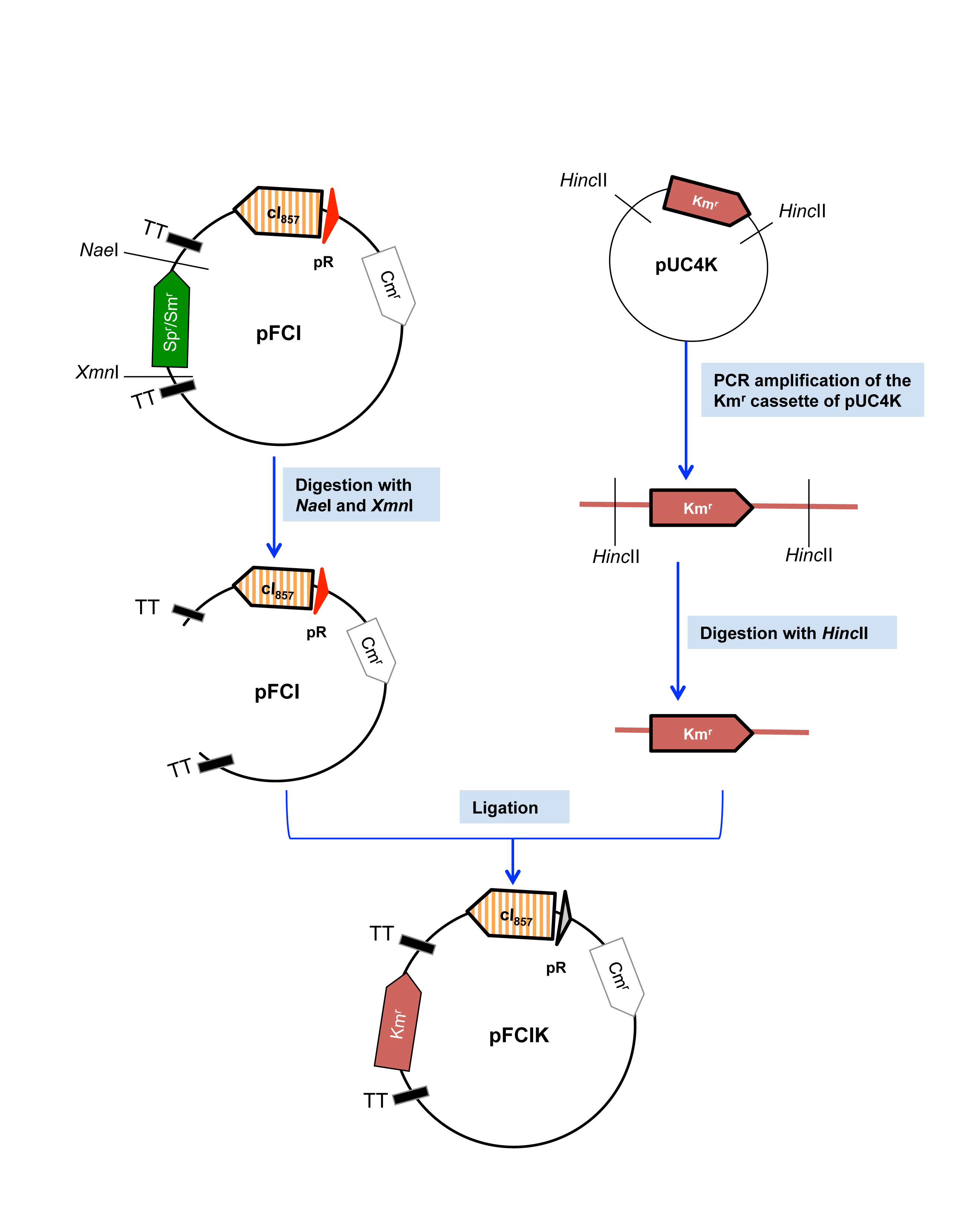

Supplement: Figure S2 — Construction of the Kmr pFC1K plasmid for temperature regulated gene expression in Synechocystis . The genes are represented by large arrows, which point in the direction of their transcription. The red triangle indicates the strong λp R promoter followed by the λcro ribosome-binding site (5′-AGGA-3′) and ATG start codon embedded within a unique NdeI restriction site (5′-CATATG-3′) for in frame-fusion of the studied protein-coding regions. They are expressed in a temperature-controlled way thanks to the λcI857 gene (hatched arrow), which encodes the temperature-sensitive repressor that tightly controls λp R. The transcription and translation stop signals (TT) preventing read-through of gene expression from the antibiotic resistance gene (Spr/Smr in pFC1 or Kmr in pFC1K) are indicated by the black bars. (TIFF) [file pone.0089372.s002.tiff]

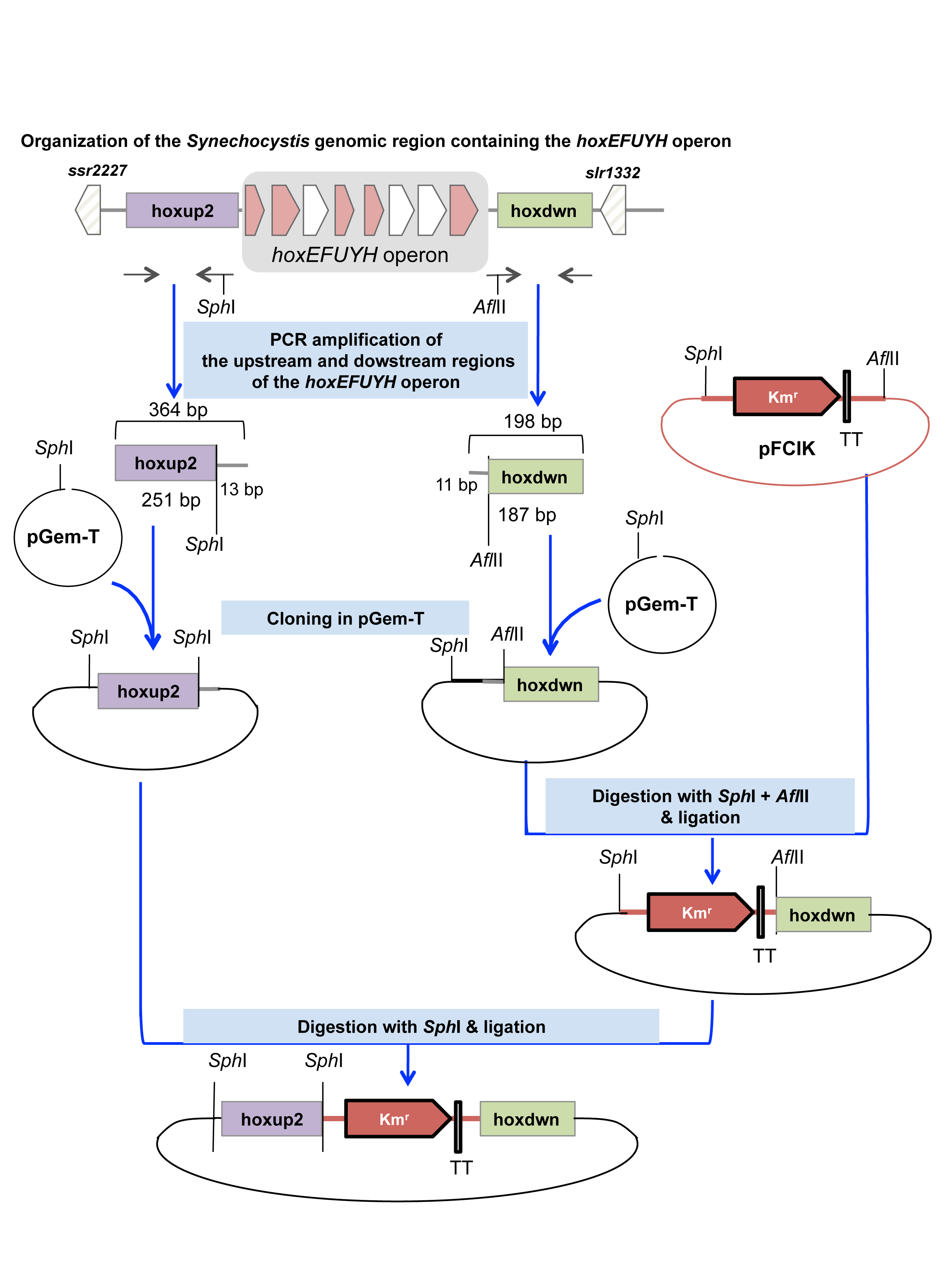

Supplement: Figure S3 — Construction of the Δ hoxEFUYH ::Kmr DNA cassette for the deletion of the Synechocystis hoxEFUYH operon, which comprises the hoxE , hoxF , sll1222 , hoxU , hoxY , ssl12420 , sll1225 and hoxH genes in that order ( Figure 1 and Figure S1). The genes are represented by white (sll1222, ssl2420 and sll1225) or pink (hoxE, hoxF, hoxU, hoxY and hoxH) boxes, which point in the direction of their transcription. The transcription terminator (TT) preventing the read-through of expression from the Kmr gene is represented by the vertical grey rectangle. The rectangles designated as hoxup2 and hoxdwn represent the DNA regions flanking the hoxEFUYH operon, which served as platforms for homologous recombinations promoting the targeted replacement of the hoxEFUYH operon by the Kmr gene. (TIFF) [file pone.0089372.s003.tiff]

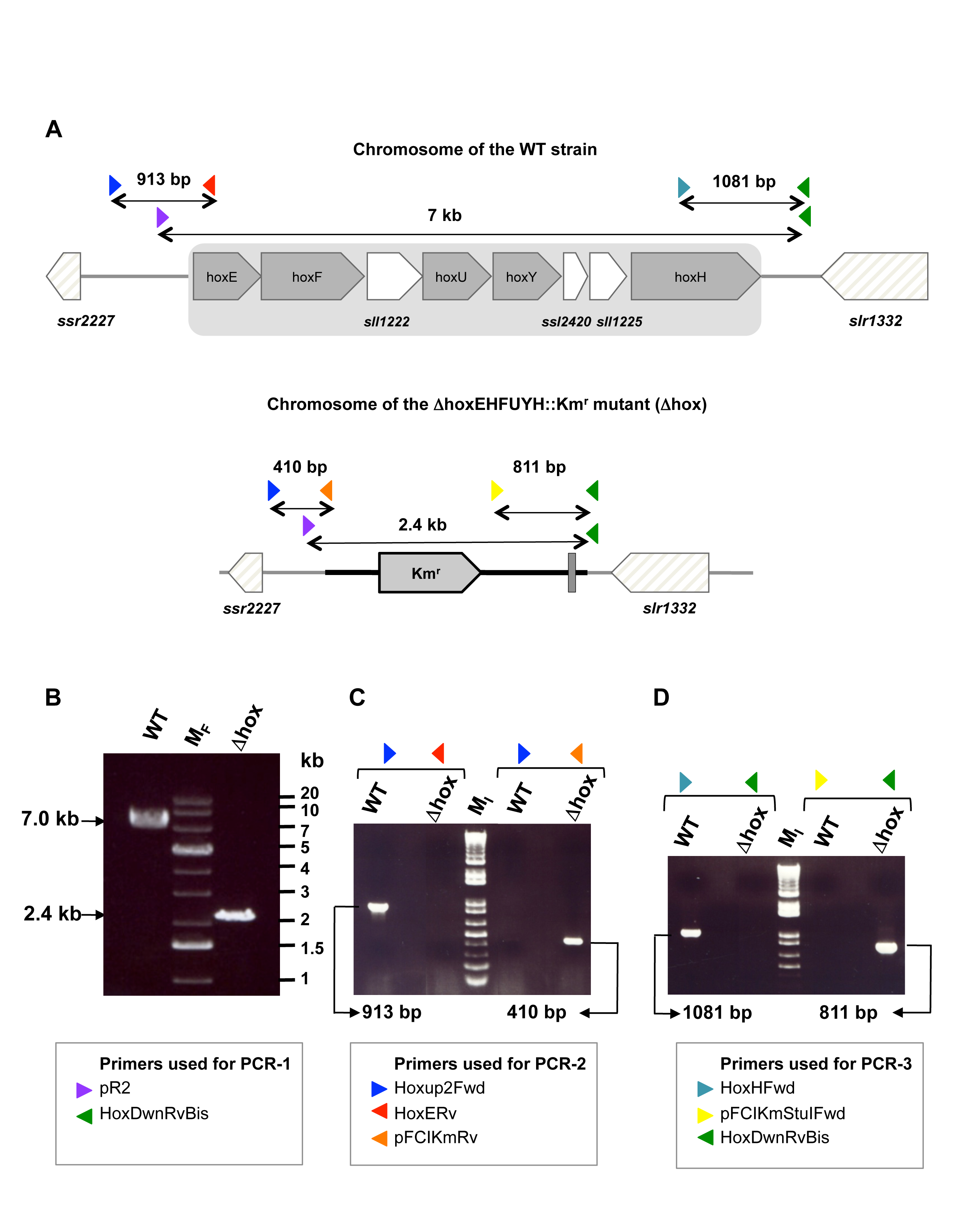

Supplement: Figure S4 — PCR verification of the Δ hoxEFUYH ::Kmr mutant showing that the replacement of the hoxEFUYH operon by the Kmr marker occurred in all copies of the polyploïd chromosome of Synechocystis . (A) Schematic representation of the hoxEFUYH operon locus in the wild-type strain (WT) and the ΔhoxEFUYH::Kmr mutant (Δhox), which harbors the Kmr marker in place of the whole hoxEFUYH operon (from 58 bp upstream of the hoxE ATG start codon, to 8 bp downstream of the hoxH TAA stop codon). The small colored triangles represent the oligonucleotides primers (Table S2) that generated the PCR DNA segments (double arrows) typical of the WT strain or the Δhox mutant. (B) UV-light image of the agarose gel showing the 7 kb and 2.4 kb PCR-1 products typical of the chromosome organization in the WT strain and the Dhox mutant growing in standard conditions. Marker (MF) = 1 Kb plus DNA Ladder (Fermentas). (C) PCR-2 and (D) PCR-3 confirmation that Δhox mutant cells contain only Dhox mutant (no WT) chromosomes. Marker (MI) = 1 Kb plus DNA Ladder (Invitrogen). (TIFF) [file pone.0089372.s004.tiff]

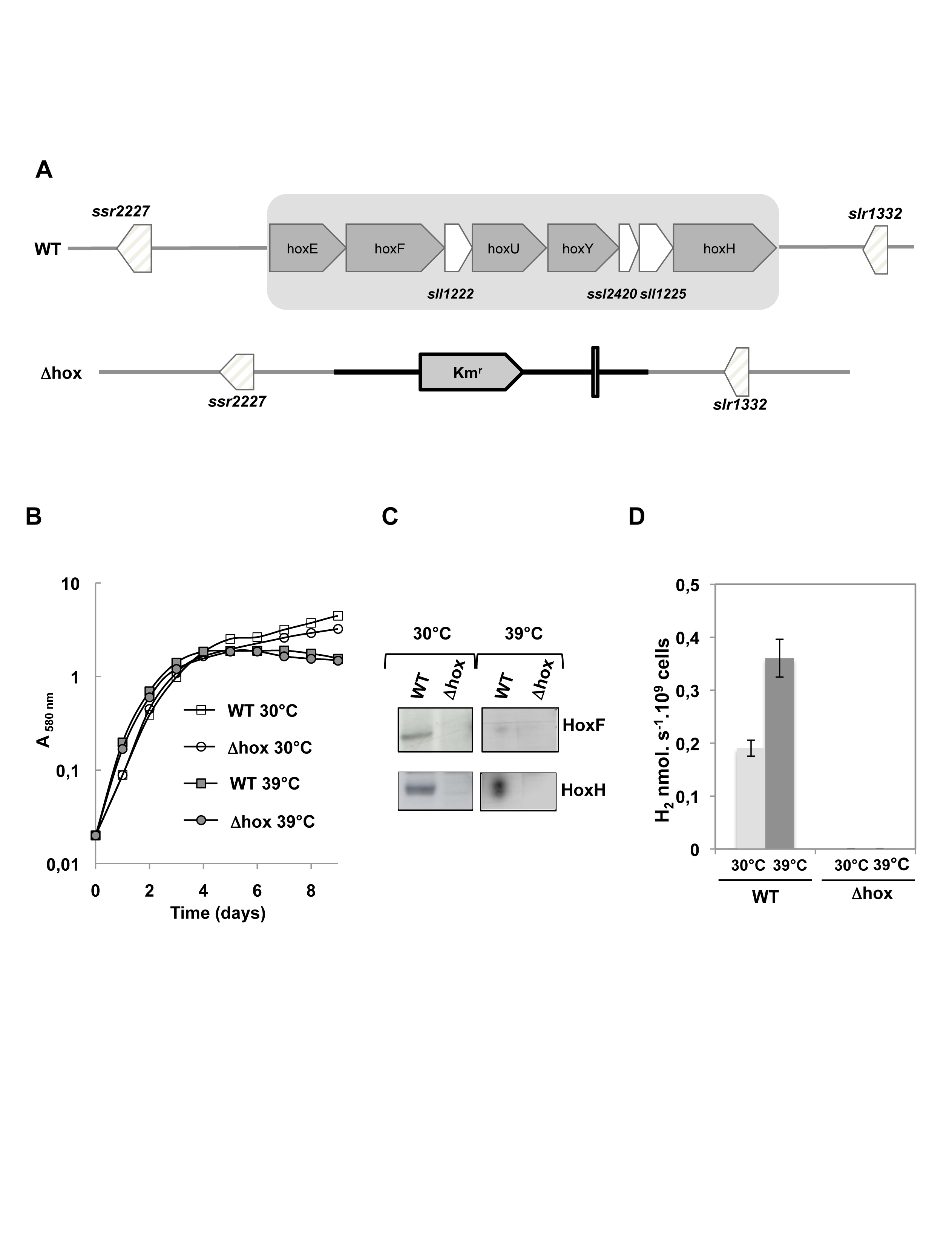

Supplement: Figure S5 — Analysis of the Synechocystis Δ hoxEFUYH ::Kmr mutant (Δhox). (A) Schematic representation of the hoxEFUYH operon locus in the WT strain or the Δhox mutant. (B) Typical growth of the WT (squares) and Δhox cells (circles) in standard conditions at either 30°C (open symbols) and 39°C (grey symbols). (C) Western blot analysis of the abundance of the HoxF and HoxH proteins in WT and Δhox cells grown at 30°C or 39°C. (D) Histograms representation of the hydrogenase activities of WT and Δhox cells grown at 30°C or 39°C. These experiment were performed three times. (TIFF) [file pone.0089372.s005.tiff]

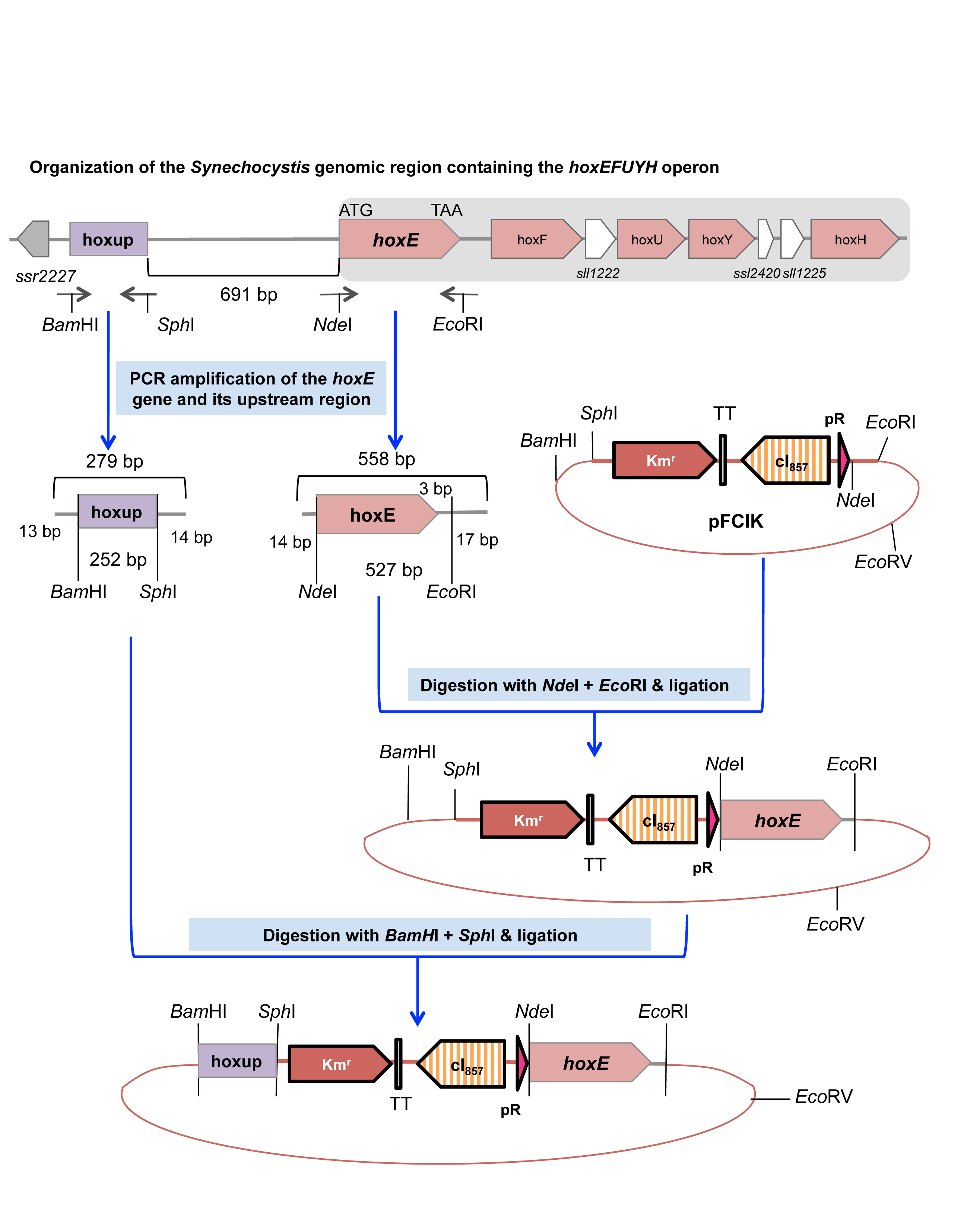

Supplement: Figure S6 — Construction of the Kmr-λ c I857-λ pR DNA cassette for temperature controlled expression of the Synechocystis hoxEFUYH operon. The genes are represented by large arrows, which indicate the direction of their transcription. The strong λ p R promoter is represented by the red triangle oppositely oriented to the lcI857 gene, which encodes the temperature-sensitive repressor that tightly controls λ p R. The transcription and translation stop signals (TT), which prevent read-through of gene expression from the Kmr marker are indicated by the vertical grey bar. The hoxup region of DNA (purple rectangle) upstream of the hoxEFUYH operon promoter and the hoxE gene served as platform for homolous recombinations, which introduced the Kmr-λ cI857-λ pR DNA cassette in place of the weak promoter [10] of the hoxEFUYH operon. (TIFF) [file pone.0089372.s006.tiff]

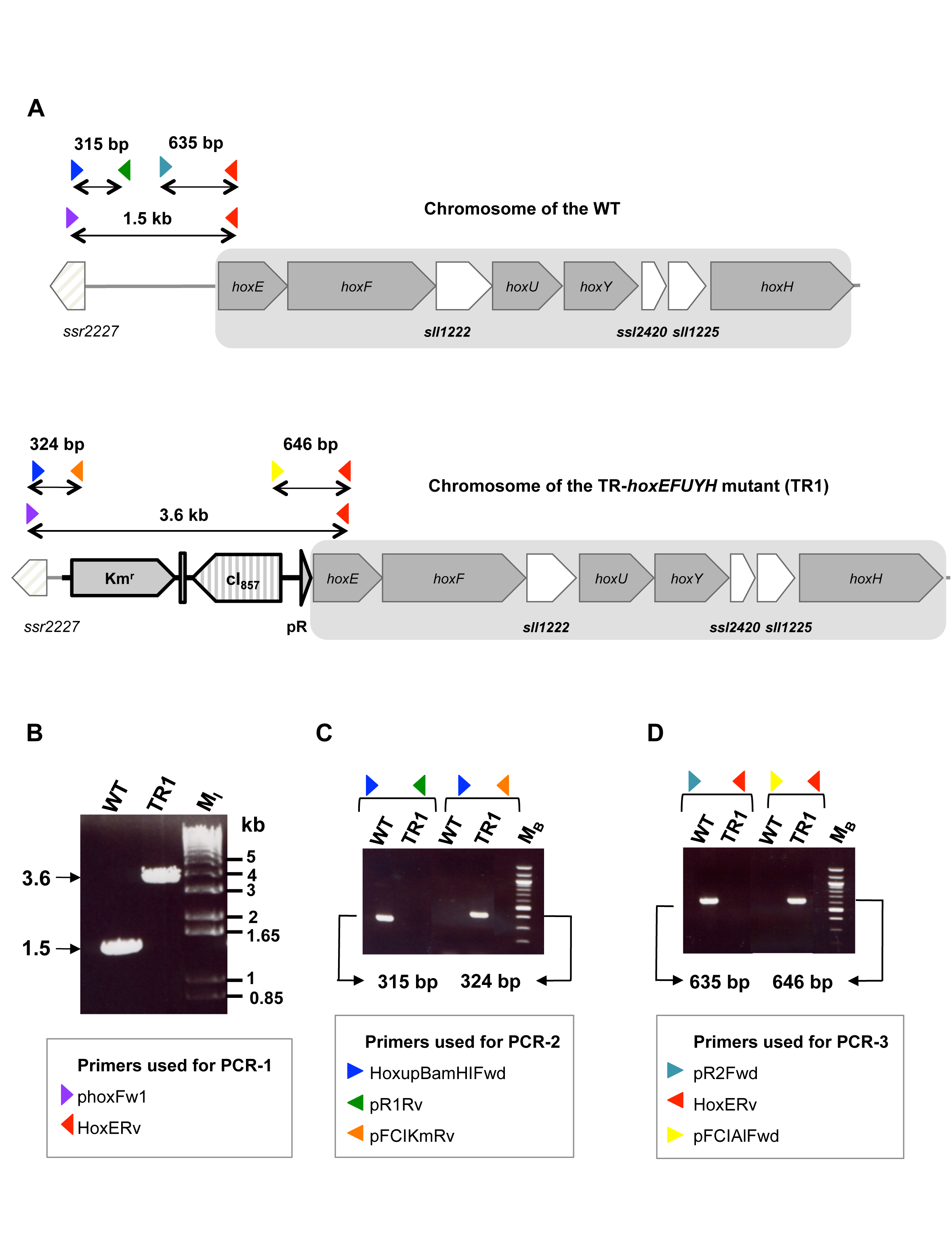

Supplement: Figure S7 — PCR verification of the Synechocystis TR- hoxEFUYH mutant (TR1) for temperature regulated high-level expression of the hoxEFUYH operon. (A) Schematic representation of the hoxEFUYH operon locus in the WT strain or the TR1 mutant, which harbors the Kmr-λ cI857-λ pR cassette in place of the natural 691 bp-long hoxEFUYH promoter region (starting from the first bp upstream of the hoxE ATG start codon). The oligonucleotides primers represented by small colored triangles (Table S2) served for the PCR verifications indicated by double arrows. (B) UV-light image of the agarose gel showing the 1.5 kb and 3.6 kb DNA products of the PCR-1 analysis of the genome of the WT strain or the TR1 mutant. Marker (MI) = 1 Kb plus DNA Ladder (Invitrogen). (C) PCR-2 and (D) PCR-3 confirmation that TR1 mutant cells contain only TR1 mutant (no WT) chromosomes. Marker (MB) = 1 Kb plus DNA Ladder (Biolabs). (TIFF) [file pone.0089372.s007.tiff]

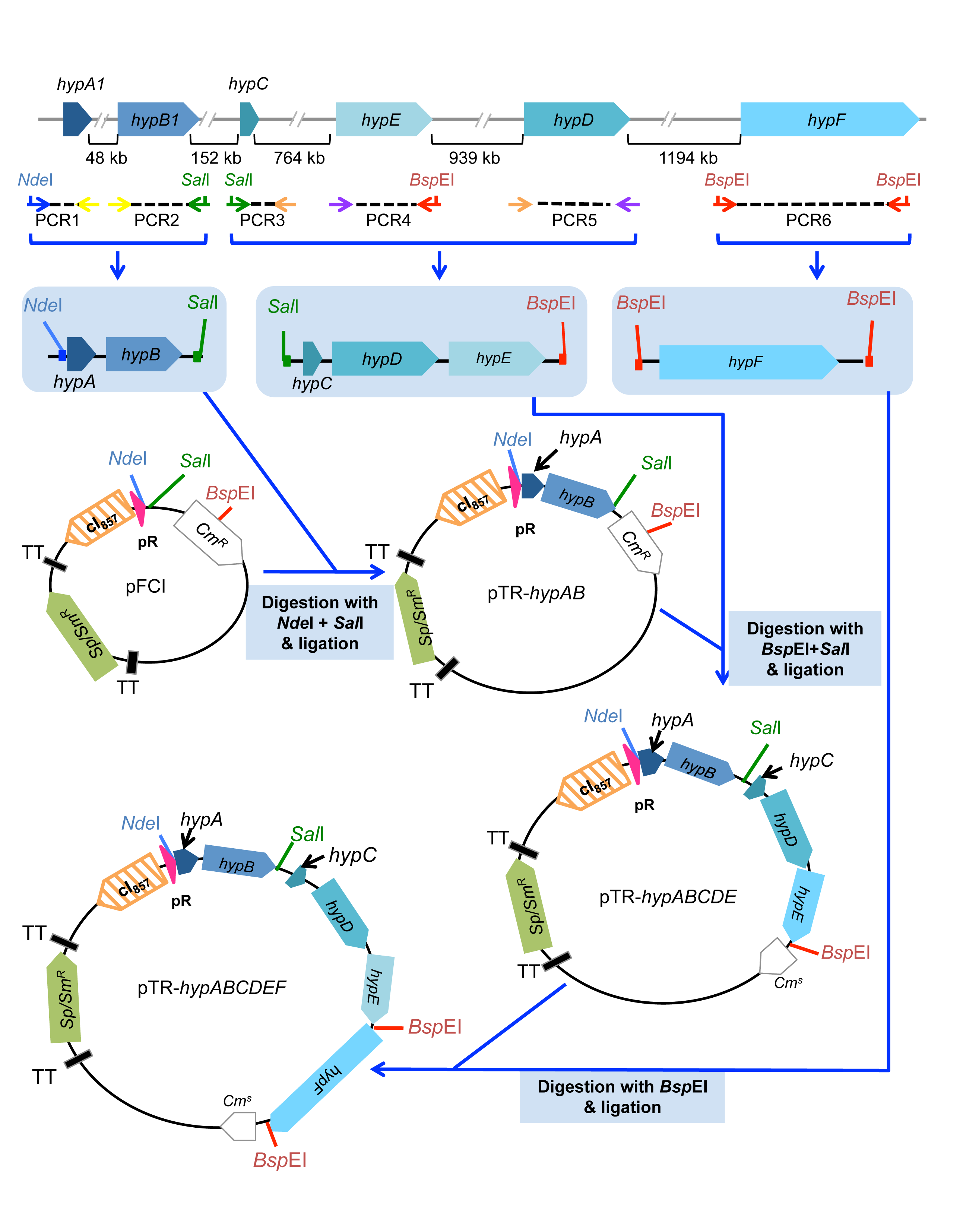

Supplement: Figure S8 — Construction of the pTR- hypABCDEF plasmid for temperature regulated expression of the Synechocystis hypABCDEF genes. For the sake of clarity, the four genes hypB1 (sll1432), hypC (ssl3580), hypE (sll1462) and hypF (sll0322) are represented oppositely to their natural orientation (Figure 1 and Figure S1). The small colored arrows indicate the position of the oligonucleotide primers used for the PCR amplification (dashed lines) and assembly (blue arrows) used for cloning the hypABCDEF genes into the pFC1 vector [5], yielding pTR-hypABCDEF. These PCR primers (Table S2) are namely: HypA1NdeIFwd (blue rightward-pointing arrow) and HypA1ASSRv (yellow leftward-pointing arrow) for PCR1; HypB1ASSFwd (yellow rightward-pointing arrow) and HypB1SalIRv (green leftward-pointing arrow) for PCR2; HypCSalIfwdbis (green rightward-pointing arrow) and HypCASSrvbis (orange leftward-pointing arrow) for PCR3; HypEASSfwd (purple rightward-pointing arrow) and HypEBspeIrv (red leftward-pointing arrow) for PCR4; HypDASSfwd (orange rightward-pointing arrow) and HypDASSrv (purple leftward-pointing arrow) for PCR5; and HypFBspeIfwdbis (red rightward-pointing arrow) and HypFBspeIrv (red leftward-pointing arrow) for PCR6. The λ p R promoter is represented by the red triangle oppositely oriented to the lcI857 repressor-encoding gene. The transcription and translation stop signals (TT) preventing read-through of gene expression are indicated by grey bars. (TIFF) [file pone.0089372.s008.tiff]

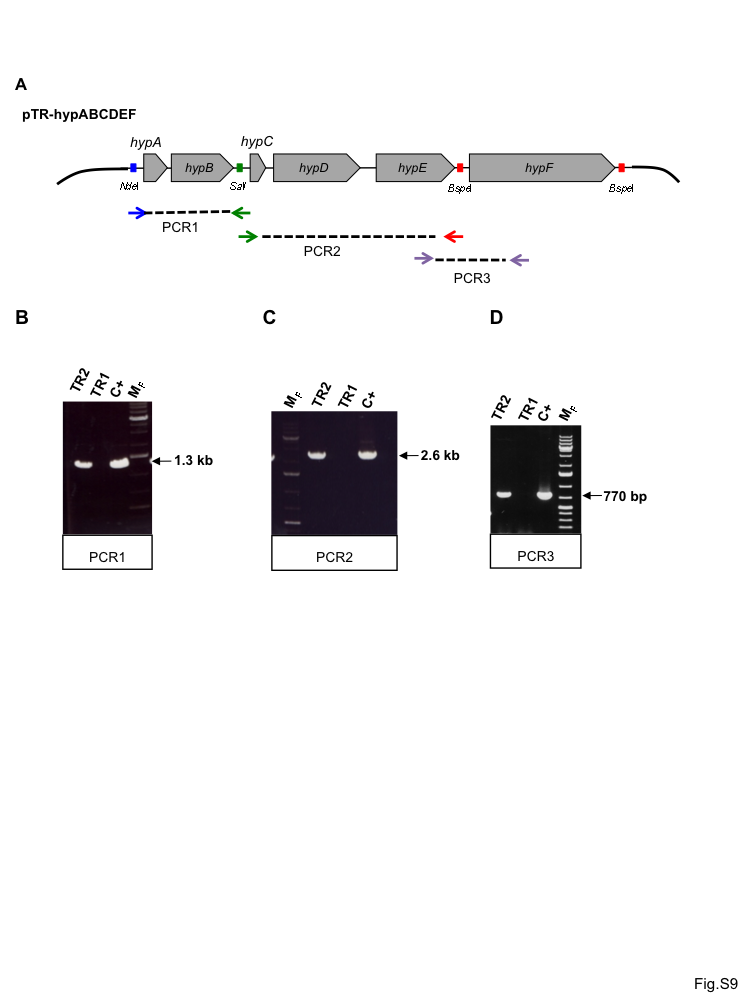

Supplement: Figure S9 — PCR verification of the pTR- hypABCDEF plasmid. (A) Schematic representation of the hypABCDEF genes (grey boxes) in the pTR-hypABCDEF plasmid replicating in E. coli (lane C+ for positive control) or in the Synechocystis mutant designated as TR-hoxEFUYH-hypABCDEF (TR2). The oligonucleotides primers (Table S2) used to generate the pTR-hypABCDEF specific DNA segments (dashed lines) of the following sizes: 1.3 kb (PCR1, panel B); 2.6 kb (PCR2, panel C) and 770 bp (PCR3, panel D) are namely: HypA1NdeIFwd (blue arrow) and HypB1SalIRv (green leftward-pointing arrow) for PCR1; HypCSalIfwdbis (green rightward-pointing arrow) and HypFBspeIFwdBis (red arrow) for PCR2; and HypDASSrv (purple leftward-pointing arrow) and HypEASSfwd (purple rightward-pointing arrow) for PCR3. Marker (MF) = 1 Kb plus DNA Ladder (Fermentas). Note that the PCR1-3 reactions can amplify only the adjacent hypABCDEF genes present in the pTR-hypABCDEF plasmid, not the chromosomal hypABCDEF genes because they are located too far away from each others (see Figure S1 and Figure S8). This explains the absence of PCR products in the negative-control Synechocystis strain TR1 (the TR-hoxEFUYH mutant), which lacks pTR-hypABCDEF. (TIF) [file pone.0089372.s009.tif]

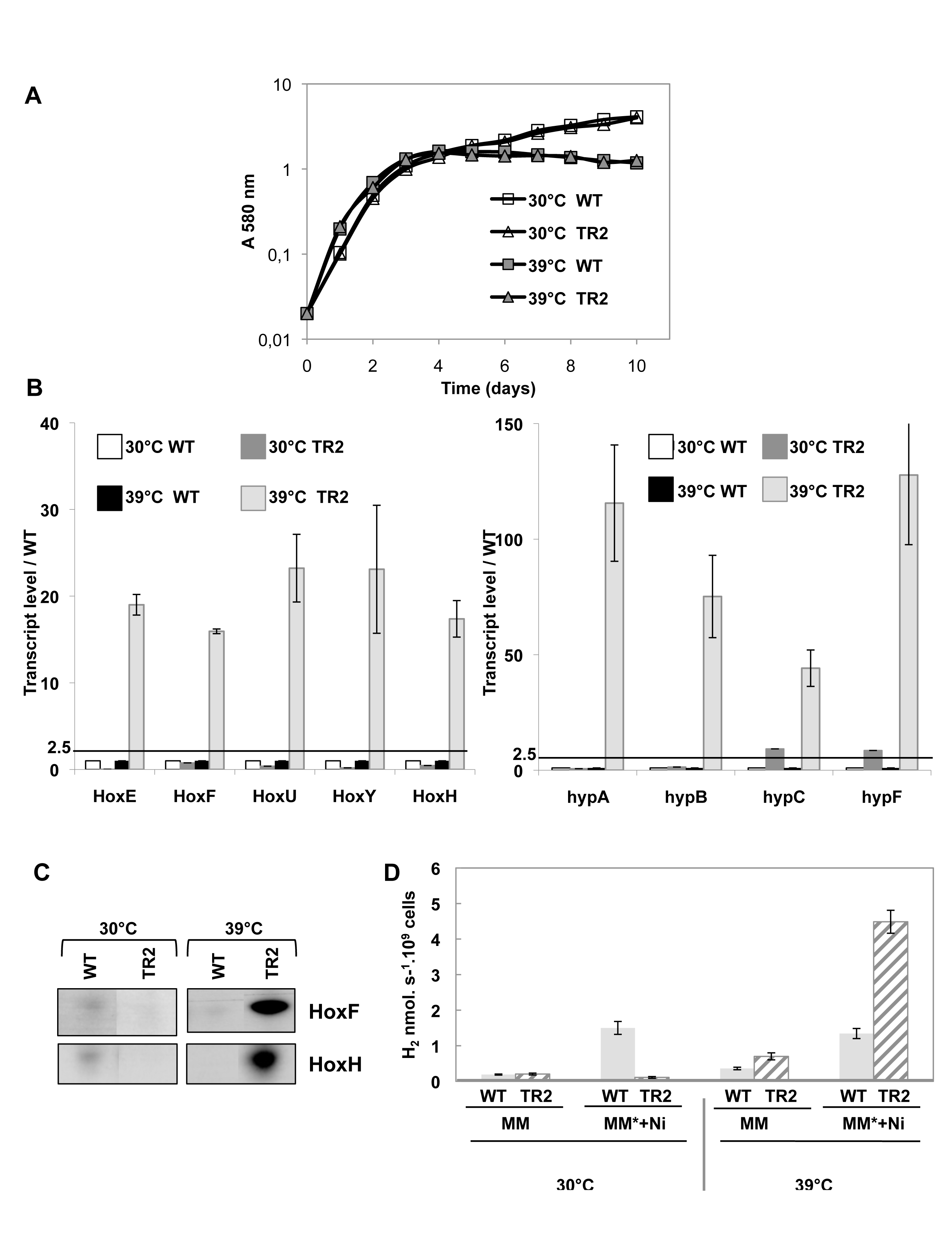

Supplement: Figure S10 — Confirmation of the temperature-controlled high-level expression of the hoxEFUYH operon and the hypABCDEF genes in the Synechocystis mutant TR- hoxEFUYH - hypABCDEF . All experiments were performed at least three times on cells grown under standard light at 30°C or 39°C. (A) Typical growth of the WT (squares) and TR-hoxEFUYH-hypABCDEF (TR2; triangles) at 30°C (white symbols) or 39°C (grey symbols). (B) Histogram plot representation of the transcript abundance (measured by Real-time quantitative PCR) of the hoxEFUYH operon (left part) and the hypABCDEF genes (right part) in WT (white bars) or TR2 (hatched bars) cells. (C) Western blot analysis of the abundance of the HoxF and HoxH proteins in WT or TR2 cells. (D) Histograms representation of the hydrogenase activities of WT (light grey), or TR2 (hatched bars) growing in standard medium (MM) or MM* (MM + 17 µM Fe) supplemented with 2.5 µM NiSO4. (TIFF) [file pone.0089372.s010.tiff]

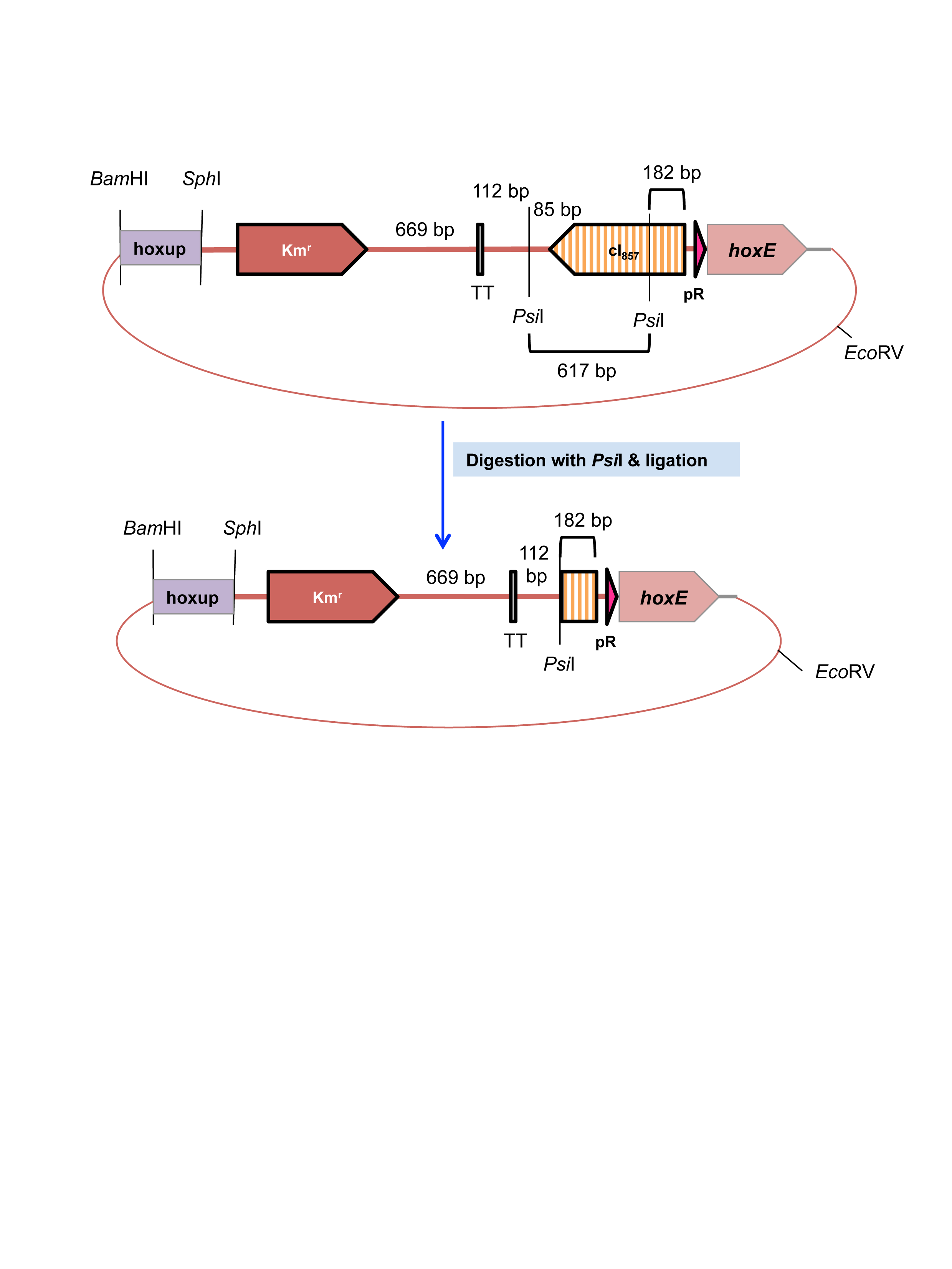

Supplement: Figure S11 — Construction of the Kmr-λ pR DNA cassette for constitutive strong expression of the Synechocystis hoxEFUYH operon. The genes are represented by large arrows, while the dark bar indicates the transcription and translation stop signals (TT), which prevent read-through of gene expression from the Kmr marker. The strong λ p R promoter is represented by the red triangle oppositely oriented to the repressor encoding-gene λ cI857, which was inactivated by PsiI restriction during the construction of the Kmr-λ pR DNA cassette. The 252 bp hoxup region of DNA upstream of the hoxEFUYH operon promoter and the hoxE gene, served as platform for homologous recombinations that introduced the Kmr-λ pR DNA cassette in place of the weak natural promoter of the hoxEFUYH operon. (TIFF) [file pone.0089372.s011.tiff]

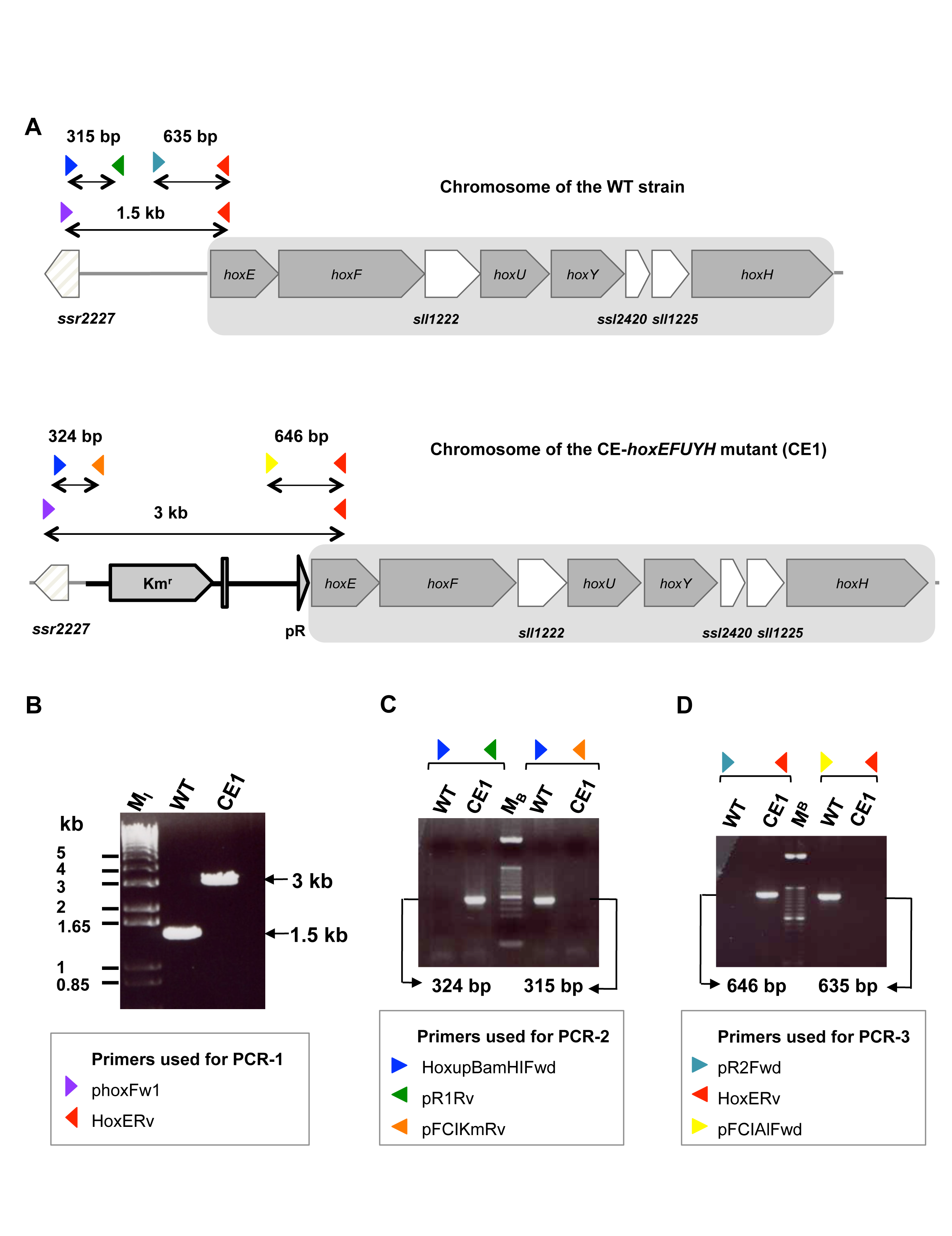

Supplement: Figure S12 — PCR verification of the CE- hoxEFUYH mutant for strong constitutive expression of the hoxEFUYH operon. (A) Schematic representation of the hoxEFUYH operon in the WT strain or the CE-hoxEFUYH mutant (CE1), which harbors the Kmr-λ pR DNA cassette in place of the natural 691 bp-long hoxEFUYH promoter region (starting from the first bp upstream of the hoxE ATG start codon). The oligonucleotides primers represented by small colored triangles (Table S2) served for the PCR verifications indicated by double arrows. (B) UV-light image of the agarose gel showing the 1.5 kb and 3.0 kb DNA products of the PCR-1 analysis of the WT strain or the CE1 mutant. Marker (MI) = 1 Kb plus DNA Ladder (Invitrogen). (C) PCR-2 and (D) PCR-3 confirmation that CE1 mutant cells contain only CE1 mutant (no WT) chromosomes. Marker (MB) = 1 Kb plus DNA Ladder (Biolabs). (TIFF) [file pone.0089372.s012.tiff]

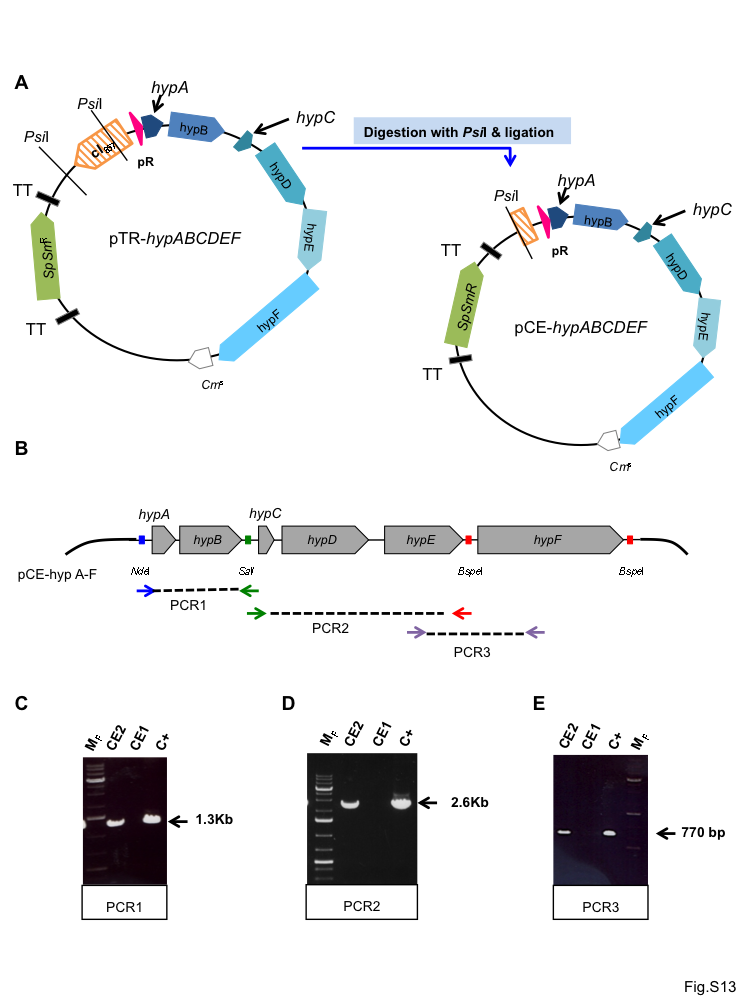

Supplement: Figure S13 — (A) Construction of the pCE-hypABCDEF plasmid for constitutive strong expression of the hypABCDEF genes. pCE-hypABCDEF was generated after the PsiI cleavage and religation of the pTR-hypABCDEF plasmid to inactivate the λcI857 repressor gene, which normally controls the strong λp R promoter (red triangle). The genes are represented by large arrows while the transcription and translation stop signals (TT) are indicated by dark grey bars. (B) Schematic representation of the hypABCDEF genes in the pCE-hypABCDEF plasmid replicating in E. coli (lane C+ for positive control) or in the Synechocystis mutant designated as CE-hoxEFUYH-hypABCDEF (CE2). The oligonucleotides primers (Table S2) used to generate the pCE-hypABCDEF specific DNA segments (dashed lines) of the following sizes: 1.3 kb (PCR1, panel B); 2.6 kb (PCR2, panel C) and 770 bp (PCR3, panel D) are namely: HypA1NdeIFwd (blue arrow) and HypB1SalIRv (green leftward-pointing arrow) for PCR1; HypCSalIfwdbis (green rightward-pointing arrow) and HypFBspeIfwdbis (red arrow) for PCR2; and HypDASSrv (purple leftward-pointing arrow) and HypEASSRwd (purple rightward-pointing arrow) for PCR3. Marker (MF) = 1 Kb plus DNA Ladder (Fermentas). Note that the PCR1-3 reactions can amplify only the adjacent hypABCDEF genes present in the pCE-hypABCDEF plasmid, not the chromosomal hypABCDEF genes because they are located too far away from each others (see Figure S1 and Figure S8). This explains the absence of PCR products in the negative-control Synechocystis strain CE1 (the CE-hoxEFUYH mutant), which lacks pCE-hypABCDEF. (TIF) [file pone.0089372.s013.tif]
