# Supplementary material for: Engineering Synechocystis PCC6803 for Hydrogen Production: Influence on the Tolerance to Oxidative and Sugar Stresses
Source: PLoS One. 2014 Feb 24;9(2):e89372. doi: 10.1371/journal.pone.0089372 (PMC3933540; doi:10.1371/journal.pone.0089372)
Supplement: Table S1 — Characteristics of the plasmids and strains used in this study. (DOCX) [file pone.0089372.s014.docx]

**Table S1.** Characteristics of the plasmids used in this study

| **Plasmids** | **Relevant feature(s)** | **Reference** |
| --- | --- | --- |
|  |  |  |
| Cloning and construction of various cassette | | |
| pGEMT | Amp^r^ AT overhang cloning vector | Promega |
| pUC4K | Source of the Km^r^ marker without TT | Pharmacia |
| pFC1 | Replicating plasmid for T°-controlled gene expression in *Synechocystis* | A |
| pFCIK | pFC1 plasmid where the Sm^r^/Sp^r^ marker was replaced by the Km^r^ gene of pUC4K to serve as a source of the Km^r^-TT-λ*cI_857_-*λ*p_R_* cassette for T°-controlled gene expression | This study  Fig S2 |
|  | | |
| Targeted deletion of *hoxEFUYH* operon in *Synechocystis* | | |
| pΔhoxEFUYH::Km^r^ | pGEMT with the Km^r^-TT-λ*cI_857_-*λ*p_R_* cassette flanked by the upstream and downstream regions of the *hoxEFUYH* operon. | This study  Fig S3 |
|  | | |
| Replacement of the *hoxEFUYH* operon promoter by the Km^r^-TT-λ*cI_857_-*λ*p_R_* cassette  for T°-regulated expression | | |
| pTR-HoxEFUYH | pFCIK with the Km^r^-TT-λ*cI_857_-*λ*p_R_* cassette flanked by the *Synechocystis* hoxup region (-943 to -691 bp upstream of the *hoxE* ATG start codon) and *hoxE* CS to serve as platform for homologous recombination mediating promoter replacement | This study  Fig. S6 |
|  | | |
| Replacement of the *hoxEFUYH* operon promoter by the Km^r^-TT*-*λ*p_R_* cassette for constitutive expression | | |
| pCE-hoxEFUYH | pTR-HoxEFUYH lacking the 617 bp region encompassing a large part (517 bp) of the λcI_857_ . | This study  Fig S11 |
|  | | |
| Temperature controlled expression of the *Synechocystis* *hypABCDEF* genes | | |
| pTR-hypABCDEF | pFCI with the  *hypABCDEF* genes expressed as a single operon under the control of the Sm^r^/Sp^r^-TT-λ*cI_857_-*λ*p_R_* cassette | This study Fig S8 |
|  | | |
| Constitutive high level expression of the *hypABCDEF* genes in *Synechocystis* | | |
| pCE-hypABCDEF | pTR-hypABCDEF lacking the 617 bp region encompassing a large part (517 bp) of the λcI_857_ . | This study  Fig S13 |

A, ([Mermet-Bouvier and Chauvat, 1994](#_ENREF_1)); CS, Protein Coding Sequence; ∆, deletion; T°, temperature; TT, transcriptional terminator

Mermet-Bouvier, P., Chauvat, F., 1994. A conditional expression vector for the cyanobacteria *Synechocystis* sp. strains PCC6803 and PCC6714 or *Synechococcus* sp. strains PCC7942 and PCC6301. Curr Microbiol. 28**,** 145-8.
