# Supplementary material for: Engineering Synechocystis PCC6803 for Hydrogen Production: Influence on the Tolerance to Oxidative and Sugar Stresses
Source: PLoS One. 2014 Feb 24;9(2):e89372. doi: 10.1371/journal.pone.0089372 (PMC3933540; doi:10.1371/journal.pone.0089372)
Supplement: Table S2 — Oligonucleotide primers used in this study. (DOCX) [file pone.0089372.s015.docx]

**Table S2** List of primers used in this study.

| **Name** | **Sequence 5’→3’** | **Purpose** | |
| --- | --- | --- | --- |
|  |  |  | |
| **Construction and PCR verification of the pFCIK** | | | |
| KmHinCFW | GGCGCTGAG**GTCGAC**CTCGTGAAGAAG | Fwd primer to amplify the Km^r^ gene of pUC4K with a *Hinc*II site | |
| KmHinCRV | ACCTGCAGGGG**GTCGAC**GGAAAGCCAC | Rev primer for the same purpose | |
|  | | | |
| **Construction and PCR verification of the** **Dhox::Km^r^  mutant** | | | |
| Hoxup2Fwd | tcaatgtacaGGATCCcaccaacaatg | Fwd primer to amplify the *hox-operon* UR starting 422 bp upstream of the *hoxE* start codon | |
| Hoxup2SphIRv | ccaggtaaattt**GCATGC**gaaagttagcc | Rev primer to amplify the *hox-operon* UR (starting 58 bp upstream of the *hoxE* start codon) and introduce a *Sph*I site | |
| HoxdwnAflIIFwd | cattcagacg**CTTAAG**aaaataccccggc | Fwd primer to amplify the *hox-operon* DR (starting 8 bp downstream of the *hoxH* stop codon) and introduce a *Afl*II site | |
| HoxDwnRvBis | ACCGGACGTCAAGTCTCGTTGACAAAA | Rev to amplify the *hox-operon* DR starting 206 bp downstream the *hoxH* stop codon | |
| pFCIKmSphIfwd | tcactgatcc**GCATGC**ccgttccata | Fwd primer to amplify the Km^r^ cassette from pFCIK with a *Sph*I site | |
| pFCIKmAflIIRv | gtgggcgggtt**CTTAAG**aattagtggtg | Rev primer to amplify the Km^r^ cassette from pFCIK with *Afl*II site | |
| HoxDwnRvBis | ACCGGACGTCAAGTCTCGTTGACAAAA | Rev primer to amplify the *hox-operon* DR (starting 206 bp downstream of the *hoxH* stop codon) and check chromosome segregation |  |
| pR2Fwd | attgtgggcaagattgtggagg | Fwd primer inside the *hox* promoter region (starting 206 bp upstream of the *hoxE* start codon) to check chromosome segregation |  |
| HoxERv | GCCAATACCGCTTCGTCATTCT | Rev primer inside of the *hoxE* CS to check chromosome segregation |  |
| HoxHFwd | GGATTGTGGACCGTTTACCA | Foward primer inside the *hoxH* CS to check chromosome segregation |  |
| pFCIKmRv | ATCCTCGGTTTTCTGGAAGGC | Rev primer to check the orientation of the Km^r^ cassette, and chromosome segregation |  |
| pFCIKmStuIFwd | gcgattc**AGGCCT**ggtatgagtcagc | Rev primer of the Km^r^ cassette harboring a *Stu*I site, used to check Km^r^ orientation and chromosome segregation |  |
|  | | |  |
| **Construction of the** **pTR-hox and pCE-hox** | | |  |

| HoxupBamHIFwd | ggcattgataat**GGATCC**ctcgtaggtctaa | Fwd primer to amplify the *hox-promoter* UR (starting 956 bp upstream of the *hoxE* start codon) with a *BamH*I site |
| --- | --- | --- |
| HoxupSphIRv | tctgggtgttgtt**GCATGC**tgatttctagg | Rev primer to amplify the *hox-promoter* UR (starting 707 bp upstream of the *hoxE* start codon) and introduce a *Sph*I site |
| HoxENdeIFwd | tttgaggatttt**CATATG**accgttgccacc | Fwd primer to amplify the *hox-promoter* DR (starting 15 bp upstream of the *hoxE* start codon) and introduce a *Nde*I site |
| HoxEEcoRIRv | tcacctgatact**GAATTC**ccgttaactgtt | Rev primer to amplify the *hox-promoter* DR (starting 9 bp upstream the *hoxE* stop codon) and introduce a *EcoR*I site |
|  | | |
| **PCR verification of chromosome segregation in TR1 and CE1 mutants** | | |
| HoxupBamHIFwd | ggcattgataat**GGATCC**ctcgtaggtctaa | Fwd primer to amplify the *hox-promoter* UR (starting 957 bp upstream of the *hoxE* start codon) with a *BamH*I site*,* and check chromosome segregation |
| phoxFw1 | GGCGACGTGCGTCCTCAAGC | Fwd primer to check chromosome segregation |
| HoxERv | GCCAATACCGCTTCGTCATTCT | Rev primer to check chromosome segregation |
| pFCIKmRv | ATCCTCGGTTTTCTGGAAGGC | Rev primer inside the Km^r^ cassette to check its orientation, and chromosome segregation |
| pFCIAlFwd | GGCGACGTGCGTCCTCAAGC | Fwd primer to check chromosome segregation |
| pR1Rv | CGAAGAAAGGAGGGTAAGTCGAACTAGTTAC | Rev primer (starting 672 bp upstream of the *hoxE* start codon) to check chromosome segregation |
| pR2Fwd | attgtgggcaagattgtggagg | Fwd primer (starting 206 bp upstream of the *hoxE* start codon) to check chromosome segregation |
|  | | |
| **Construction and PCR verification of the pTR** | | |
| HypA1NdeIFwd | cccagagttaat**catATG**CACGAAGTTAGTCTG | Fwd primer to amplify *hypA1* (starting 15 bp upstream of its start codon) with a *Nde*I site |
| HypA1ASSRv | TTAATGTTATGAcccatgcTCAACTCAGTTC | Rev primer to amplify *hypA1* (starting 15 bp upstream its stop codon) for PCR assembly with with *hypB1* |
| HypB1ASSFwd | GGTCATAACATTAAtatttccgcccg**AGGAGG** | Fwd primer to amplify *hypB1* (starting 41 bp upstream of its start codon) along with *hypA1* downstream region by PCR assembly |
| HypB1SalIRv | gtcactgcc**GTCGAC**tttttccttcaactc | Rev primer to amplify *hypB1* (starting downstream of its stop codon) with a *Sal*I site |
| HypCSalIFwdbis | ctgactta**GTCGAC**aattcctcc**AGGAGG**tcctcccATGTGTC | Fwd primer to amplify *hypC* (starting 36 bp upstream of its start codon) with a *Sal*I site and a SD |
| HypCASSrvbis | CCAAGGTTAACGctctattcttaactctaggaa**ttA**AACTCCC | Rev primer for PCR assembly amplification of *hypC* (starting 10 bp upstream its stop codon) along with *hypD* UR |
| HypDASSfwd | gagCGTTAACCTTGGaatcagtt**AGGAGG**gttt | Fwd primer for PCR assembly amplification of *hypD* (starting 38 bp upstream of its start codon) along with the *hypC* DR |
| HypDASSRv | TGCAGGATGTAAttgtctgatcccagcaaaac | Rev primer for PCR assembly amplification of *hypD* (starting 1 bp before *hypC* stop codon) along with the *hypE* UR |
| HypEASSFwd | gaaTTACATCCTGCAacaaag**AGGAGG**gctg | Fwd primer for PCR assembly amplification of *hypE* (starting 34 bp upstream of its start codon) along with the *hypD* DR |
| HypEBspeIRv | cagtctagggct**TCCGGA**ttggagtcCTAAC | Rev primer to amplify *hypE* (starting 5 bp upstream its stop codon) with a *Bspe*I site |
| HypFBspeIFwd bis | cata**TCCGGA**tatttaacagtattaa**AGGAGG**tagaataATG | Fwd primer to amplify *hypF* (starting 39 bp upstream of its start codon) with a *Bspe*I site |
| HypFBspeIRv | tttggtta**TCCGGA**gttgagTTAACAGATATATTGCCG | Rev primer to amplify *hypF* (starting 18 bp before its stop codon) with a *Bspe*I site |
| HypELeft | GCCTGTGAACTGTTGGGTTT | Fwd primer of *hypE* to verify *hypF* orientation |
| HypFRight | AGTCGGTACATTGGCGAAAC | Rev primer of *hypE* to verify *hypF* orientation |
| **RT qPCR analysis** | | |
| HoxE Left | GCCCATCCTAGTGGAGACAA | *hoxE* Fwd primer starting 42 bp upstream of its ATG start codon |
| HoxE Right | GGTAAAAAGTCGCCACTCCA | *hoxE* Rev primer starting 301 bp upstream of its stop codon |
| HoxF Left | TGAAATGACCCCAGAGGAAG | *hoxF* Fwd primer starting 478 bp downstream of its ATG start codon |
| HoxF Right | ATACCTTCGAGGATGCGATG | *hoxF* Rev primer starting 948 bp upstream of its stop codon |
| sll1222 Left | TGCTATGGCTATCCCCTCTG | *sll1222* Fwd primer starting 325 bp downstream of its ATG start codon |
| sll122 Right | AGCTCCCTTGGTTTTCCATT | *sll1222* Rev primer starting 145 bp upstream of its stop codon |
| HoxU Left | GTTTGTGCATGGTGGAAGTG | *hoxU* Fwd primer starting 142 bp downstream of its start codon |
| HoxU Right | ATTCACAGTTGCCGTTAGCC | *hoxU* Rev primer starting 406 bp downstream of its stop codon |
| HoxY Left | CTCAAGGAATACCCGGACAA | *hoxY* Fwd primer starting 155 bp downstream of its start codon |
| HoxY Right | AGAACCGGATCGCTACCTTT | *hoxY* Rev primer starting 249 bp upstream of its stop codon |
| ssl2420 Left | CCCCAGAGGAGCAAAAGTTA | *ssl2420* Fwd primer starting 45 bp downstream of its start codon |
| ssl2420 Right | TCTTGGGTTCTTTCCTCACG | *ssl2420* Rev primer starting 48 bp upstream of its stop codon |
| sll1225 Left | TGTCCGACTTCTTGTTGGGTA | *sll1225* Fwd primer starting 68 bp downstream of its start codon |
| sll1225 Right | AGCAATTTGGGCTTGAGTGT | *sll1225* Rev primer starting 248 bp upstream of its stop codon |
| HoxH Left | CTGCGCCGTTTAATGAATTT | *hoxH* Fwd primer starting 293 bp downstream of its start codon |
| HoxH Right | CACTGACCAAGCAGAGTGGA | *hoxH* Rev primer starting 938 bp upstream of the *hoxY* stop codon |
| HypA1 Left | TACGGTTTGCGTTTGAAGTG | *hypA1* Fwd primer starting 130 bp downstream of its start codon |
| HypA1 Right | GTTCCAACTGTTTGCCATCC | *hypA1* Rev primer starting 43 bp upstream of the *hypB1* stop codon |
| HypB1 Left | GGGCTATTTCCAGGCTAAGG | *hypB1* Fwd primer starting 239 bp downstream of its start codon |
| HypB1 Right | GAGACGTTGGGCATCGTTAT | *hypB1* Rev primer starting 476 bp upstream of its stop codon |
| HypC Left | CTCCGATCCCCTGTTACTGA | *hypC* Fwd primer starting 44 bp downstream of its start codon |
| HypC Right | CTCCCATTTCTGCCAAGTCT | *hypC* Rev primer starting 25 bp upstream of its stop codon |
| HypD Left | GGGAAAGGGCCTATCAACAT | *hypD* Fwd primer starting 618 bp downstream of its start codon |
| HypD Right | TCGACTGGCTCAAACACTTG | *hypD* Rev primer starting 333 bp upstream of its stop codon |
| HypE Left | GCCTGTGAACTGTTGGGTTT | *hypE* Fwd primer starting 809 bp downtream of its start codon |
| HypE Right | CCCAAGGTTTGTGCACTTTT | *hypE* Rev primer starting 102 bp upstream of the *hypD* stop codon |
| HypF Left | TCCGCCTGCTTAACAGAACT | *hypF* Fwd primer starting 341 bp downstream of its start codon |
| HypF Right | AGTCGGTACATTGGCGAAAC | *hypF* Rev primer starting 1846 bp upstream of its stop codon |

The restriction sites are written in blue while the Shine-Dalgarno sequences are written in red.

bp: base pair; CS: protein coding sequence; DR: downstream region; Fwd: fwd; Rv: rev; UR: upstream region
