# Supplementary material for: Engineering Synechocystis PCC6803 for Hydrogen Production: Influence on the Tolerance to Oxidative and Sugar Stresses
Source: PLoS One. 2014 Feb 24;9(2):e89372. doi: 10.1371/journal.pone.0089372 (PMC3933540; doi:10.1371/journal.pone.0089372)
Supplement: Table S3 — List of the Hox and Hyp hydrogenase proteins detected in Synechocystis WT strain or CE2 mutant grow in standard conditions using LC-MS/MS (Orbitrap) or LCMS/MS(Q-Exactive) techniques. ND: Non Detected. (DOCX) [file pone.0089372.s016.docx]

**Table S3**

List of the Hox and Hyp hydrogenase proteins detected in *Synechocystis* WT strain or CE2 mutant growing under standard conditions. ND: Non Detected.

| **Hox and hyp proteins detected in soluble proteins from WT or CE2 cells**  **using LC-MS/MS (Orbitrap) technique** | | | | | | |
| --- | --- | --- | --- | --- | --- | --- |
| **Protein ID in Cyanobase** | **Name** | **Mass (kDA)** | **Number of spectra in WT** | **Number of spectra**  **in CE2** | **Unique**  **peptides** | **Sequence coverage (%)** |
| sll1220 | HoxE | 18.7 | ND | 5 | 4 | 47 |
| sll1221 | HoxF | 57.6 | ND | 32 | 20 | 54 |
| sll1222 |  | 24.1 | ND | 9 | 7 | 44 |
| sll1223 | HoxU | 26.1 | ND | 16 | 11 | 56 |
| sll1224 | HoxY | 19.9 | ND | 2 | 2 | 23 |
| sll1226 | HoxH | 52.8 | ND | 37 | 23 | 65 |
| slr1675 | HypA1 | 12.7 | ND | 6 | 3 | 42 |
| sll1432 | HypB1 | 31.1 | ND | 8 | 5 | 26 |
| ssl3580 | HypC | 8 | ND | ND | ND | ND |
| slr1498 | HypD | 40 | 0* | 2* | 2* | 5* |
| sll1462 | HypE | 36,4 | ND | ND | ND | ND |
| sll0322 | HypF | 85.2 | ND | 3 | 3 | 4 |
|  |  |  |  |  |  |  |
| **Hox and hyp proteins detected in total protein extracts from WT or CE2 cells**  **using LC-MS/MS (Q-Exactive) technique** | | | | | | |
| **Protein ID in Cyanobase** | **Name** | **Mass (kDA)** | **Number of spectra in WT** | **Number of spectra**  **in CE2** | **Unique**  **peptides** | **Sequence coverage (%)** |
| sll1220 | HoxE | 18.7 | 1 | 4 | 4 | 39 |
| sll1221 | HoxF | 57.6 | 1 | 36 | 25 | 55 |
| sll1222 |  | 24.1 | 0 | 6 | 5 | 30 |
| sll1223 | HoxU | 26.1 | 0 | 14 | 9 | 53 |
| sll1224 | HoxY | 19.9 | ND | ND | ND | ND |
| sll1226 | HoxH | 52.8 | 0 | 24 | 23 | 54 |
| slr1675 | HypA1 | 12.7 | 0 | 4 | 4 | 48 |
| sll1432 | HypB1 | 31.1 | 0 | 4 | 7 | 26 |
| ssl3580 | HypC | 8 | ND | ND | ND | ND |
| slr1498 | HypD | 40 | 0 | 2 | 4 | 16 |
| sll1462 | HypE | 36,4 | ND | ND | ND | ND |
| sll0322 | HypF | 85.2 | ND | ND | ND | ND |
